# Characterization of a novel and active temperate phage vB_AbaM_ABMM1 with antibacterial activity against *Acinetobacter baumannii* infection

**DOI:** 10.1038/s41598-023-38453-7

**Published:** 2023-07-13

**Authors:** Meity Mardiana, Soon-Hian Teh, Yun-Chan Tsai, Hsueh-Hui Yang, Ling-Chun Lin, Nien-Tsung Lin

**Affiliations:** 1 Institute of Medical Sciences, Tzu Chi University, No. 701, Sec. 3, Zhongyang Rd., Hualien, 97004 Taiwan; 2 Division of Infectious Diseases, Department of Internal Medicine, Hualien Tzu Chi Hospital, Buddhist Tzu Chi Medical Foundation, No. 707, Sec. 3, Zhongyang Rd., Hualien, 97004 Taiwan; 3 Department of Life Sciences, Tzu Chi University, No. 701, Sec. 3, Zhongyang Rd., Hualien, 97004 Taiwan; 4 Department of Medical Research, Hualien Tzu Chi Hospital, Buddhist Tzu Chi Medical Foundation, No. 707, Sec. 3, Zhongyang Rd., Hualien, 97004 Taiwan; 5 Master Program in Biomedical Sciences, School of Medicine, Tzu Chi University, No. 701, Sec. 3, Zhongyang Rd., Hualien, 97004 Taiwan

**Keywords:** Microbiology, Molecular biology

## Abstract

*Acinetobacter baumannii* is an opportunistic pathogen that significantly causes hospital-acquired infections. Due to its multidrug resistance, treating infections caused by this pathogen is challenging. Recently, phages have gained attention as a potential alternative to antibiotics in treating bacterial infections. While lytic phages are preferred in therapy, the use of temperate phages for this purpose has received less attention. This study characterized a novel temperate phage vB_AbaM_ABMM1 (ABMM1) with antibacterial activity toward *A. baumannii.* ABMM1 adsorbs quickly, has short latent periods, and is relatively stable at various temperatures and neutral pH. ABMM1 has an icosahedral head and a contractile tail. It has a 75,731 kb circular permuted dsDNA genome containing 86 gene products with 37.3% G + C content and a mosaic arrangement typical of temperate phages. Genomic analysis confirmed that ABMM1 does not have antibiotic-resistance genes or virulence-related factors. The packaging strategy was predicted in silico, suggesting that ABMM1 represents a headful phage. Only truncated ABMM1 prophage was detected and has similarity in the genome of several *A. baumannii* strains. Despite its ability to integrate into the host chromosome, the high MOI of ABMM1 (MOI 10) effectively killed the host bacterial cells and reduced the fatality rate of bacterial infection in the zebrafish model. These findings indicate that ABMM1 can be an alternative treatment for *A. baumannii* infection.

## Introduction

*Acinetobacter baumannii* is an opportunistic pathogen that has emerged as a significant cause of hospital-acquired infections worldwide. The most frequent sites of infection are the urinary tract, respiratory tract, bloodstream, surgical wounds, and other diseases^1^. This pathogen is renowned for resisting multiple drugs through several mechanisms, including beta-lactamase production, efflux pumps, reduced membrane permeability, and modification of antibiotic target sites. Consequently, treating infections caused by this pathogen is challenging^2^. In recent years, phages as potential alternatives to antibiotics for treating bacterial infections have gained significant attention. Phages are viruses that infect bacteria and have been used to treat bacterial infections for years. In fact, certain phage preparations have been produced in accordance with medical prescriptions, enabling personalized therapy for individual patients. Furthermore, these phage products have undergone rigorous processing to adhere to the standards set forth by Good Manufacturing Practice (GMP)^3,4^. In combating *A. baumannii* infection, some phages have been isolated and characterized by their strengths and limitations^5–9^. The characterization of the phages infecting *A. baumannii* and their use of in vivo model play important role in advancing the field of phage therapy. For example, the use of phage Bϕ-R2096, a lytic phage which specifically causes the lysis of carbapenem-resistant *A. baumannii*, was characterized in detail in vitro*,* and its effect was also evaluated using *Galleria mellonella* and a mouse model of acute pneumonia^10^. Another effectiveness of the use of phage in infecting *A. baumannii* was also shown by phage vB-AbauM-Arak1, a lytic phage with a broad host range that significantly improved burn infection in rat models^11^. While lytic phages have been studied and preferred in therapy, the use of temperate phages for this purpose has received less attention.

Temperate phages are a type of phage capable of having both lytic and lysogenic cycles, meaning they can either replicate within the host cell, leading to the lysis of the bacterium and subsequent phage release, or integrate their genome into the host chromosome and remain dormant (lysogenic cycle). The integration is mediated by specific recombination events between the phage genome and the host chromosome. Temperate phage genome can be divided into several key regions with distinct functions; the regulatory region, which contains genes that regulate the decision between the lysogenic and lytic cycles, lysogeny control region, which contains genes responsible for maintaining the prophage state within the host chromosome, replication region, structural genes, and the lysis genes, which are responsible for the lytic cycle that leads to the lyse of the host cell and the release of new phage particles^12^. Temperate phages are abundant in nature and common in pathogen genomes, yet their roles are still unclear. In short, temperate phages potentially benefit bacteria; they play a role in the Horizontal Gene Transfer of bacteria through transduction, as a source of genetic variation for bacteria evolution, and act as weapons of bacterial competition^13^. Despite the benefits that temperate phages may confer upon their bacterial hosts, it is essential to note that they can also have detrimental effects. Temperate phage integration reduces bacterial pathogenicity in *Bordetella bronchiseptica* by disrupting pilin gene expression, leading to a noticeable decline in virulence in the parental strain^14^. Despite the current knowledge gaps, investigations into the relationship between temperate phages and their host continue to provide valuable insight and interest.

Many *A. baumannii* phages are characterized; most are lytic, and only less is temperate^6–9^. This study isolated a temperate phage from the environmental sample, showing antibacterial activity against *A. baumannii.* The isolated phage was characterized, and its genome was sequenced. We investigated the ability of this phage to infect *A. baumannii* cells in vitro, and evaluated its therapeutic potential in a zebrafish model of *A. baumannii* infection. Our results indicated that this temperate phage has the potential to be used as an effective antibacterial agent against *A. baumannii* infections, and its use may provide a promising alternative to antibiotics. This study also serves as a valuable addition to the existing literature by enhancing our understanding of the role and function of temperate phage in this particular context.

## Results

### Phage isolation and morphology

**Figure 1:**
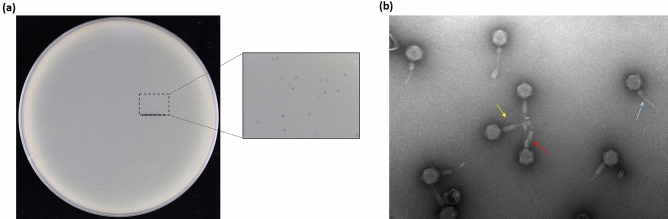
ABMM1 morphology. (**a**) Plaque morphology of ABMM1 on *A. baumannii* TV2199 lawn; (**b**) Electron micrograph of ABMM1 phage. The head is approximately 94 nm and the contractile tail is approximately 118 nm in size, measured by ImageJ software from 4 measurements^16^. Red arrow indicates a phage with a fully relaxed tail, yellow arrow indicates a partially contracted tail, and blue arrow indicates a fully contracted tail.

Phage against *A. baumannii* strain TV2199 was isolated from wastewater around Buddhist Tzu Chi Hospital and named vB_AbaM_ABMM1 (shortened to ABMM1). ABMM1 morphology was observed by TEM (Fig. 1). The phage had small and turbid plaques; typical feature of temperate phage as lytic phages are commonly associated with clear plaques^15^. The phage head was icosahedral with a dimension of ~ 94 nm and had a contractile tail of ~ 118 nm. This phage was then assigned to the class *Caudoviricetes* with a myovirus morphology.

### Phage host range analysis

We determined the host range of ABMM1 by spot test using 38 clinical isolates of *A. baumannii* and 2 ATCC strains (Supplementary Table S1). The results showed that only 16 strains were susceptible to ABMM1 (40%). ABMM1 exhibits a narrower host range when compared to our previously reported phage (TCUP2199), which could infect 85% of those strains^7^.

### Biological characteristics of ABMM1

**Figure 2:**
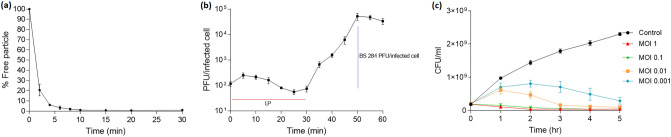
Biological characteristics of ABMM1. (**a**) Adsorption kinetics of ABMM1. Approximately 80% of phage particles had adsorbed onto the cells after 2 min, and nearly 98% after 30 min; (**b**) One-step growth curve of ABMM1. The latent period was approximately 30 min and the burst size was 284 PFU/infected cell. *LP* latent period, *BS* burst size; (**c**) ABMM1 ability to reduce bacterial growth at different MOI. Note that a higher MOI can minimize bacterial growth better than a lower MOI. Untreated bacterial growth curve was assigned as a control. These experiments were performed in triplicate, and the data were shown in the mean ± SEM.

The adsorption rate of ABMM1 onto TV2199 was determined and the result showed that about 80% of the phage particles adsorbed to host cells within 2 min and almost 98% after 30 min (Fig. 2a). One-step growth experiment was done as shown in Fig. 2b. The result showed that ABMM1 had a latent period of about 30 min and the burst size was approximately 284 PFU/infected cell after the latent period. Different MOI of ABMM1 (0.001, 0.01, 0.1, and 1) were used to infect the host TV2199 in the log phase. The growth curves showed that the number of bacteria decreased gradually after adding the ABMM1 and the rate of decrease was also proportional to the MOI (Fig. 2c).

### ABMM1 is stable at 37 °C and pH 7

**Figure 3:**
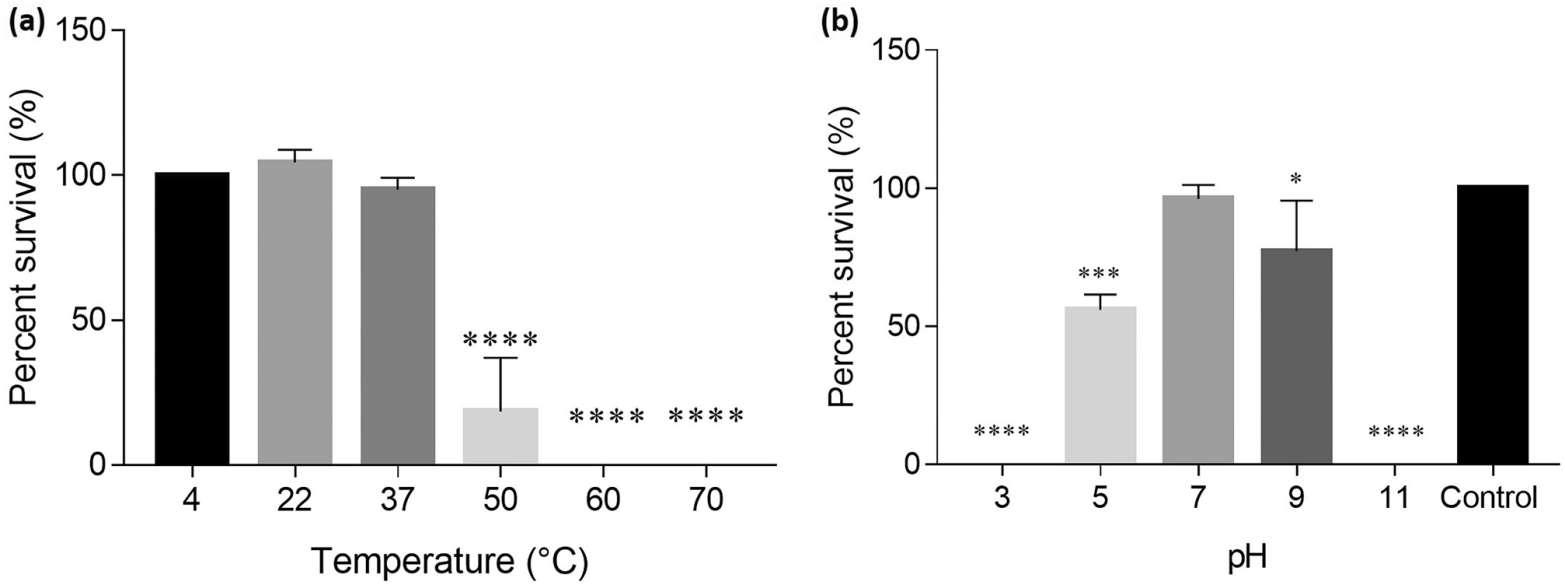
Temperature and pH stability of ABMM1. (**a**) ABMM1 stability was assessed by subjecting it to different temperatures for 1 h and its impact on the initial loaded phage was evaluated; (**b**) ABMM1 stability in different pH. Phage titer was compared with the initial load (control) titer. Asterisks indicate significant differences (*P ≤ 0.05; ***P ≤ 0.001; ****P ≤ 0.0001).

The stability of ABMM1 in various environments was checked to determine its optimum storage condition and for phage therapy. The titer of ABMM1 was stable at 4–37 °C and significantly decreased at 50 °C. No plaques were observed after ABMM1 was kept at 60 and 70 °C for 1 h (Fig. 3a). ABMM1 titer remained the same with the initial load (control) at pH 7, while partial loss of infective activity was observed at pH 5 and pH 9 compared to the initial load (control). The absence of plaque formation at pH 3 and 11 indicates that ABMM1 is unable to tolerate extreme acidity or alkalinity conditions (Fig. 3b).

### Whole genome analysis of ABMM1

**Figure 4:**
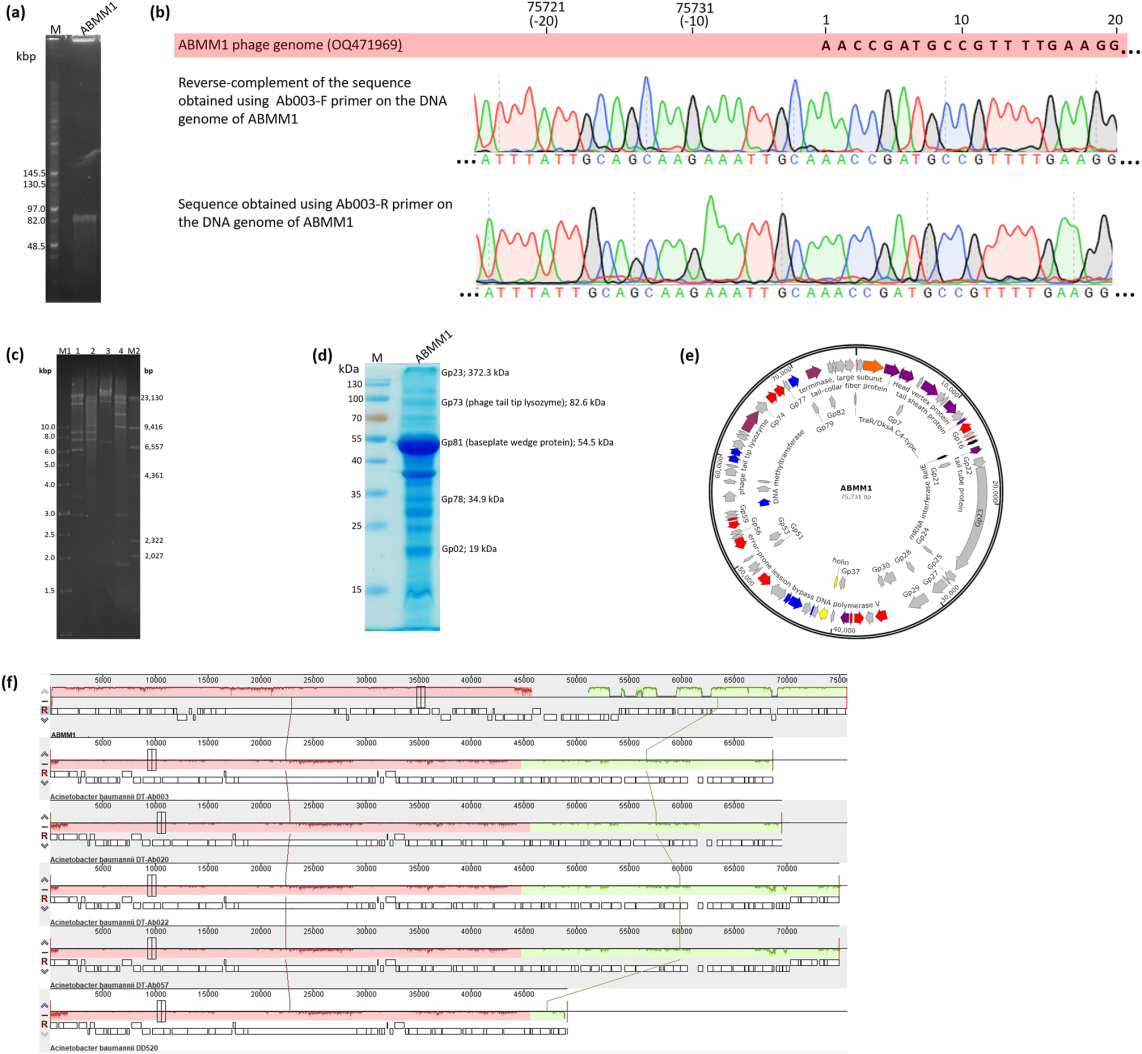
ABMM1 genome and protein analysis and annotation. (**a**) ABMM1 genome size based on PFGE. Lane M: Midrange PFG Marker (New England BioLabs, Ipswich, MA, USA). The original gel is presented in Supplementary Fig. S7; (**b**) ABMM1 genome termini verification. The first row shows the rightward terminus nucleotide sequence of ABMM1 genome (first 20 bases). Row 2 and 3 show Sanger-based sequencing read chromatograms corresponding to both termini, peaks are colored based on the base call and their height represents the relative quality of the called base; (**c**) Restriction enzyme analysis of ABMM1. ABMM1 genomic DNA digested with *Ava*I (band sizes are 32,871 bp, 18,330 bp, 8233 bp, 7340 bp, 6069 bp, 2888 bp) (lane 1), *Xho*I (band sizes are 38,940 bp, 21,218 bp, 8233 bp, 7340 bp) (lane 2), *Bgl*I (band sizes are 49,868 bp, 19,152 bp, 6711 bp) (lane 3), and *Nhe*I (band sizes are 29,045 bp, 19,463 bp, 12,965 bp, 9482 bp, 2885 bp, 1852 bp, 39 bp) (lane 4). Lane M1, 1-Kb DNA ladder and lane M2, Lambda digested with *Hind*III ladder (New England Biolabs). The original gel is presented in Supplementary Fig. S8; (**d**) ABMM1 protein analysis by 12% SDS-PAGE. Lane M: Prestained protein marker, Plus (Protech, Taipei, Taiwan). The original gel is presented in Supplementary Fig. S9; (**e**) Genome annotation of ABMM1. Orange color represents packaging proteins, purple color represents tail-related proteins, blue color represents replication and transcription-related proteins, red color represents lysogenic-related proteins, yellow color represents lysis-related proteins, and grey color represents hypothetical proteins. The map was drawn by Snapgene Viewer version 6.1.2. (https://www.snapgene.com/snapgene-viewer); (**f**) Genome-wide alignment of ABMM1 between the two direct repeats at the end and corresponding regions in the *A. baumannii* strain DT-Ab003 (CP050916) (nt 2,977,165–3,045,803); DT-Ab020 (CP050911) (nt 3,027,508–3,097,037); DT-Ab022 (CP050907) (nt 3,002,032–3,077,019); DT-Ab057 (CP050904) (nt 2,960,758–3,035,745); and DD520 (CP075321) (nt 2,959,037–3,008,168) by Mauve alignment. Boxes with identical color represent local are a region of collinear sequence among all 6 genomes. Non box indicates the absence of homologous sequence within the region. The blocks on the top row indicate the gene products were facing the same direction on the DNA, while the blocks on the bottom row are in inverse orientation.

PFGE result estimated the genome size of ABMM1 to be approximately 82 kb (Fig. 4a), while the accurate genome size of 75,731 bp was established based on sequencing. ABMM1 has a circular permuted genome with a 127-bp repeat present at both ends, as confirmed by the reads spanning over the predicted termini (Fig. 4b). To validate its circularity and compare its arrangement to the in silico prediction, experimental restriction enzyme digestion was performed. The result of the experimental restriction enzyme digestion of ABMM1 exhibited a perfect match with in silico restriction digestion fragments (Fig. 4c).

To check whether ABMM1 has cohesive ends, *Ava*I digested phage DNA was analyzed in an agarose gel after heat treatment at 80 °C and cooled either rapidly on ice or slowly. Under slow cooling condition, the cohesive-ended fragments will anneal to one another and be visible as larger bands, but under fast cooling, the two terminal bands do not have time to aneal to one another^17,18^. The result shows no different band pattern between the ones with rapid and slow cooling, meaning ABMM1 does not have cohesive ends (Supplementary Fig. S1). We also checked the pattern using the other three restriction enzymes, but it also showed no difference between the two conditions.

The G + C content of ABMM1 is 37.3%, lower than some *A. baumannii* complete genomes available in the database (Supplementary Table S2). RAST successfully annotated 86 predicted genes from the ABMM1 genome, of which 66 gene products (76.7%) were encoded on the same DNA strand, while the remaining 20 gene products (23.3%) were on the opposite strand. ATG was the predominant initiation codon [93% (80/86)]. Only 5 gene products began with GTG, and 1 began with TTG. The predominant termination codons were TAA [63.5% (55/85)], followed by TGA [23.5% (20/85)] and TAG [12.9% (11/85)]. No tRNA genes were found in the ABMM1 genome, suggesting phage protein synthesis relies on host tRNA. No antibiotic resistance genes or virulence factors were found in the genome according to ResFinder and VirulanceFinder, respectively. Moreover, a significant proportion (96.5%) of gene products exhibited hits to annotated genes present in the *A. baumannii* genomes, as determined through BLASTP analysis. However, HHpred analysis revealed that these gene products also displayed hits to corresponding proteins associated with other sequenced and annotated phages. Through in silico analysis, it was determined that the ABMM1 genome encompasses distinct functional gene modules. First, the genes that encode structural proteins include tail-collar fiber protein (Gp04), baseplate wedge subunit (Gp05), tail protein (Gp06), head vertex protein (Gp09), tail sheath proteins (Gp12; Gp14), tail tube protein (Gp20), baseplate tail tube protein (Gp36), and baseplate wedge protein (Gp81). Two of the gene products were confirmed by LC–MS/MS (Fig. 4d) along with 4 hypothetical proteins. Second, the genes encoding gene products associated with lysogenicity were scattered throughout the genome. Gp48 was identified as an integrase that belongs to the tyrosine recombinase family, with an N-terminal arm DNA-binding domain and a C-terminal catalytic domain (Supplementary Fig. S2). The Xre family transcriptional regulator (Gp32), RecA family protein (Gp54), some repressors (Gp35; Gp57; Gp58), and antirepressors (Gp15; Gp17; Gp76) are also potentially involved in lysogenic/lysis decisions. Additionally, ABMM1 contained a Type II toxin-antitoxin system (Gp75), which might participate in stabilizing the lysogen and controlling phage production^19^. Phage replication and transcription proteins were encoded by *g19*; *g42*; *g44*; *g61*; *g68*; and *g69*. The phage genome also encodes gene products involved in lysis, including holin and putative lysin (Gp39 and Gp40, respectively). The Gp39 was predicted to be holin as it is relatively small (95 amino acids) and had two transmembrane domains that belong to type II holin^20^ predicted by Deep TMHMM^21^. No gene encoding spanin was identified in the genome. The gene *g73*, located far from the other lysis-related genes, is predicted to encode a phage tail tip lysozyme (Gp73) far downstream from the previous lysis-related genes. Overall, the ABMM1 genome was arranged in a mosaic organization where genes having similar functions are dispersed throughout the genome (Fig. 4e). The annotation of the complete genome is available in Supplementary Table S3.

According to BLASTP, most gene products of ABMM1 show similarity to gene products encoded by genes located in the genomes of *A. baumannii* strains. In addition, it was shown that ABMM1 was classified as a temperate phage by PhageAI analysis. Therefore, we performed the Mauve alignment of the top 5 *A. baumannii* genomes most similar to the ABMM1 genome. We compared the putative prophages of the bacterial genomes, which contain the whole unique sequence portion and both ends of the two direct repeats. The result revealed that ABMM1 has a longer sequence size than the corresponding regions of the bacterial genomes, suggesting that loss of some ABMM1 genomic fragment occurred and only truncated ABMM1 prophage exists on the chromosome of *A. baumannii*, indicating that the prophages is not functional. About 64.8 kb of the ABMM1-like region encodes some structural genes and lysogeny-related genes. However, this fragment lacks the integrase gene and part of replication genes, thereby resulting a gap in the alignment (Fig. 4f). Besides, a genome alignment was performed by Easyfig, which revealed that the truncated ABMM1 prophage in the *A. baumannii* genome was inverted at both the 5′ and 3′ ends of ABMM1, that is, two 127-bp repeat ends of ABMM1 merged into one at the ABMM1-like region of *A. baumannii* genome, just at the inverted junction of the two fragments (Supplementary Fig. S3). This finding validated the circularization of ABMM1 through the recombination of its terminally redundant ends, followed by integration into the host chromosome, thereby producing prophage^22^.

Furthermore, we searched for the existence of prophage on the genomes of these 5 strains using Prophage Hunter (http://pro-hunter.genomics.cn/). It demonstrated that the genomes of these strains harbored numerous prophage candidates but that the specific fragment resembling ABMM1 was absent (Supplementary Table S4). This result might be attributed to the loss of several phage-associated genes, such as integrase and some replicative genes. Thus, it just counts as a truncated prophage.

### ABMM1 can form a prophage pattern after infecting the host

**Figure 5:**
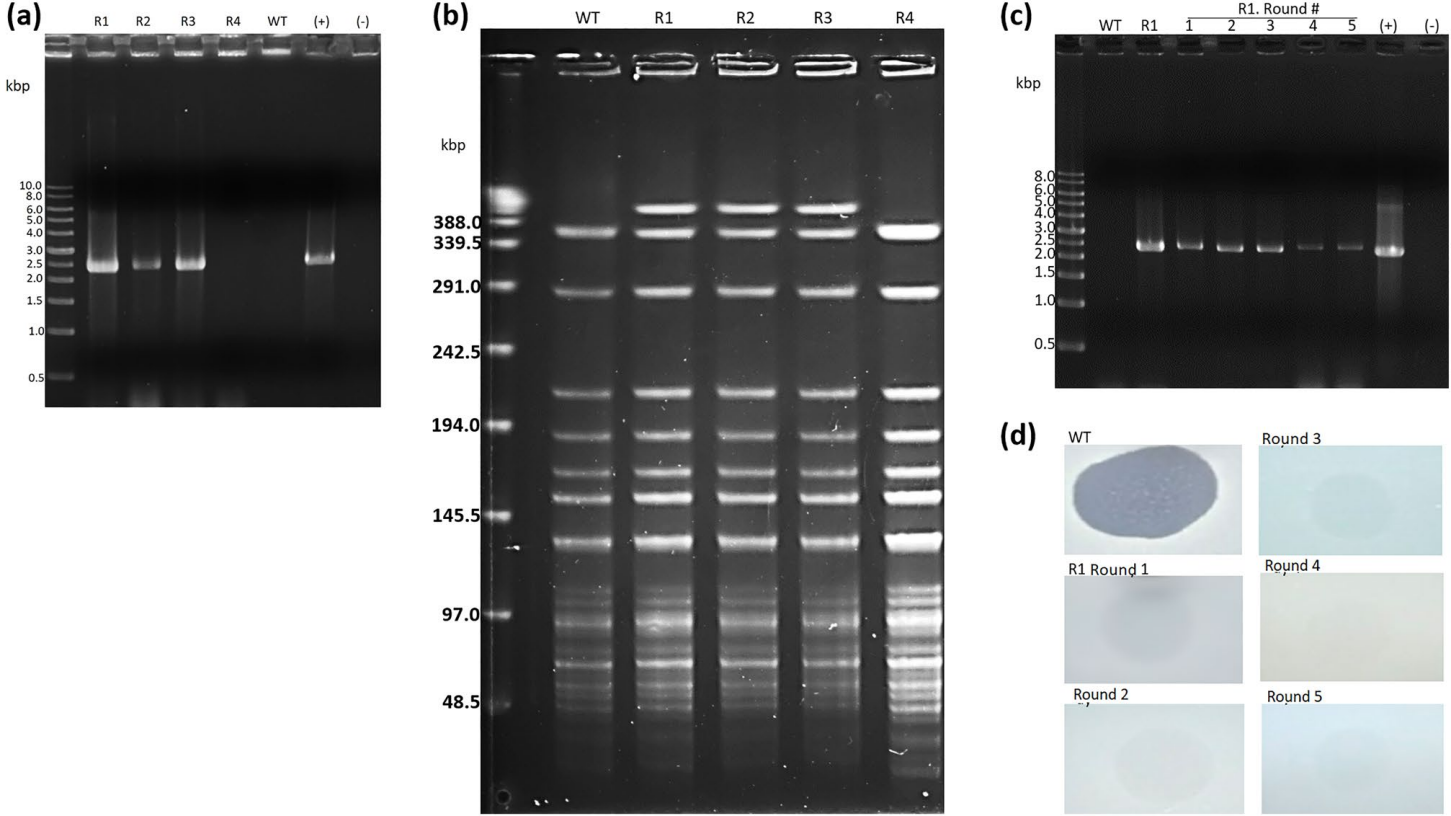
ABMM1 can be lysogenized to TV2199. (**a**) PCR amplification of ABMM1 Gp73 (phage tail tip lysozyme) from phage-resistant strain genomes, Bio-1Kb™ as DNA ladder (New England BioLabs). The original gel is presented in Supplementary Fig. S10; (**b**) PFGE profiles of phage-resistant strains digested with *Apa*I (Fermentas), Lambda PFG ladder as the marker (New England BioLabs). The original gel is presented in Supplementary Fig. S11; (**c**) PCR amplification of ABMM1 Gp73 from R1 strains after subculture for 5 rounds of passage. The original gel is presented in Supplementary Fig. S12; (**d**) Spot test of ABMM1 in R1 lawn for 5 rounds of passage compared to WT lawn. R1-R4 indicates the number of phage-resistant colonies, WT indicates parental strain TV2199, (+) indicates ABMM1 DNA as the positive control, and (−) indicates no template used in PCR as the negative control.

After an extended co-culture period of ABMM1 and the host, colonies resistant to the phage emerged. Since lysogen-related genes were found in the ABMM1 genome sequence and it was predicted as a temperate phage by PhageAI, we further examined the presence of ABMM1 in the genome of the infected host. Therefore, we randomly picked phage-resistant colonies derived from the parental wild-type strain TV2199. These colonies were then employed as PCR templates to investigate the potential formation of a prophage by ABMM1. The designated primers for this analysis were designed to target the sequences of *g48* (integrase) and *g73* (phage tail tip lysozyme). In 3 of the 4 colonies, the 2.3-kb fragment was amplified by *g73*-specified primers, indicating that there was ABMM1 phage in the genomes of these 3 phage-resistant colonies (Fig. 5a). The same result was also seen when the genome was amplified by *g48-*specified primers (Supplementary Fig. S4). Furthermore, PFGE was also performed to determine if the genome of ABMM1 had been integrated into the genome of phage-resistant colonies instead of existing as a plasmid. We used *Apa*I, which could not cut the ABMM1 genome, to digest the chromosomal DNA of phage-resistant colonies. The result revealed the presence of an additional band exceeding 388 kbp in size compared to the wild-type strain, which may indicate that the genome of ABMM1 was integrated into the chromosome of phage-resistant colonies (Fig. 5b). However, for the remaining colony R4, its PCR and PFGE detection results were not similar to those of the other three colonies; that is, PCR amplified no band, and no extra band was found in PFGE analysis. To confirm the reasons for becoming phage-resistant, we carried out several rounds of subculture on the R4. The results showed that R4 lost its resistance to ABMM1 (Supplementary Fig. S5) after 5 rounds of subcultures, suggesting that the phage receptor encoding gene(s) could be under phase variation, allowing the mutation to revert. To check that the lysogenization of ABMM1 remained stable, we performed PCR with *g73* primers to confirm the ABMM1 genome was still present in the R1 genome after five streaking cycles from a single colony. We also assessed ABMM1 infectivity by conducting a spot test in R1 and parental wild-type strain TV2199 lawns. This finding demonstrated the persistent presence of the *g48* and *g73* amplicons within the R1 genome, despite the inability of ABMM1 to infect R1 even after 5 rounds of passage (Fig. 5c,d). In addition, in order to know whether other ABMM1-insensitive *A. baumannii* clinical strains also contain the prophage type of ABMM1 in the genome, we performed colony PCR detection on 23 clinical isolates using *g73-*specific primer and no amplicons were observed (Supplementary Fig. S6).

### Phylogenetic tree indicated ABMM1 has distant similarity with other phages and has headful packaging strategy

**Figure 6:**
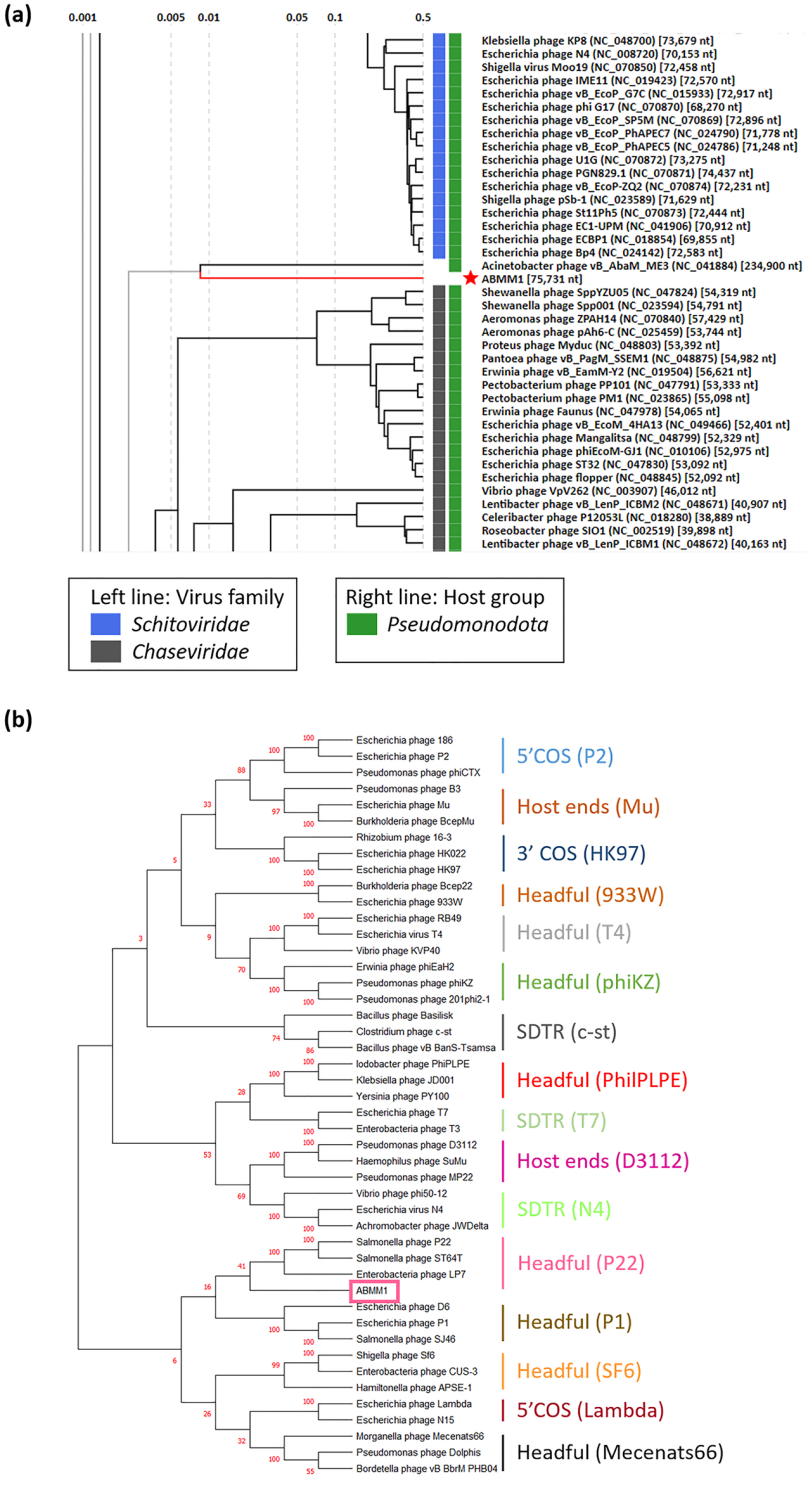
Phylogenetic tree analysis of ABMM1. (**a**) Viral proteomic tree of ABMM1 against all reference phages in the database generated by VIPtree version 3.6 (https://www.genome.jp/viptree/). The figure are focusing on the closer-related phages of ABMM1. (**b**) The prediction of ABMM1 packaging strategy based on TerL of ABMM1 and the dataset from previous study. MUSCLE aligned the amino acid sequences and MEGA X was used to construct the tree based on Neighbor-joining method with 1000 bootstraps.

In light of ABMM1’s high similarity to bacterial host genomes based on BLASTN, we endeavored to ascertain its relationship to other phages through phylogenetic tree analysis. A wide-genome proteomic tree validated that ABMM1 does not have close similarity with other phages. It was only clustered together and has distant relatedness with *Acinetobacter* phage ME3 (NC_041884), which is a unique jumbo phage that has a mosaic genome arrangement and is classified as the only member of the genus *Metrivirus* with an unclassified family (Fig. 6a). To further predict the packaging strategy, we utilized MEGA X to produce a phylogenetic tree of ABMM1 and other phages based on the terminase large subunit (TerL), a conserved protein in phages using a similar approach from a previous study^23^. The resulting tree showed that ABMM1 (NC_041884) clustered together with the headful (P22) genome packaging strategy employing phages but formed a single clade, suggesting that its packaging strategy was unique as it has some distinctions from P22-like phages (Fig. 6b).

### ABMM1 is effective in treating *A. baumannii*-infected zebrafish

**Figure 7:**
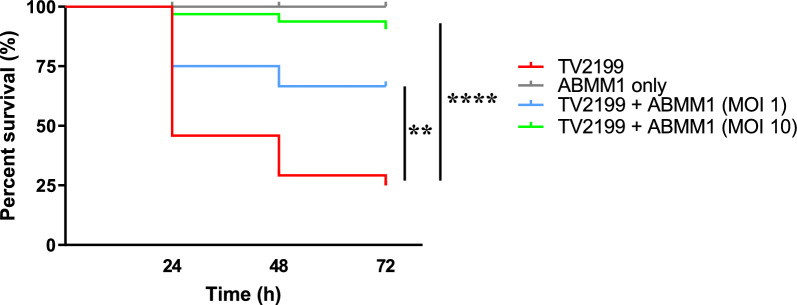
ABMM1 therapy in zebrafish bacterial infection model. Each group consisting of 8 zebrafish and subjected to 4 different conditions as follow: injected with TV2199 cells only (10^6^ PFU/20 µl, red line), injected with TV2199 cells followed by ABMM1 treatment with MOI 1 and 10 (blue and green line, and injected with ABMM1 only (pink line). The significance of the difference between groups was performed by Mantel–Cox test (**P ≤ 0.005; ****P ≤ 0.0001).

The phage therapy effect of ABMM1 was tested by injecting ABMM1 into zebrafish with various MOIs after they were challenged with TV2199 for 30 min. After 24 h, almost 50% of fish challenged with TV2199 died, while approximately 75% and 100% were alive for fish treated with ABMM1 MOI 1 and 10, respectively. About 75% of fish challenged with TV2199 died after 72 h, whereas approximately 75% were rescued after being treated with ABMM1 MOI 1. Almost all fish were still alive after MOI 10-treatment. This experiment also showed that ABMM1 was safe to use as therapy, as after 72 h, all fish were still alive after receiving only phage injection (Fig. 7).

## Discussion

This study presents the isolation and characterization of temperate phage ABMM1 and its genomic analysis. ABMM1 belongs to the class *Caudoviricetes* with a myovirus morphology as it has a tail and an icosahedral capsid as confirmed by TEM^24^. We later checked its relatedness to all phages in the VIPtree database. The result indicated that ABMM1 had no close relatedness to other phages. The only one that had a distant relationship was with *Acinetobacter* phage ME3, which was also known as singleton^6,25^. ABMM1 formed tiny and turbid plaques on bacterial lawns, a common feature of temperate phages. In addition to its ability to integrate into the bacterial host genome as a temperate phage, ABMM1 exhibited a rapid absorption rate, a large burst size, stability under neutral pH and temperature conditions, and effective antibacterial activity both in vitro and in vivo.

The whole genome sequence of ABMM1 possessed high similarity (96.62%) with the genomes of several *A. baumannii* strains. It could integrate into the infected host after co-cultivating with the host for a period of time. However, Prophage Hunter did not identify any prophage candidates resembling full ABMM1 in the databases. Thus, we considered ABMM1 a novel and active temperate phage from the environment. Viral TerL with the same packaging mechanism tends to form clades in the phylogenetic tree. Phylogenetic analysis successfully predicted the packaging strategy of ABMM1, as it clustered with other P22-like phages, albeit forming a single clade. This finding suggests that ABMM1 may represent an evolutionary unique headful genome packaging system. In a headful phage, approximately 103% of the whole genomic DNA is incorporated into the phage head from a covalent concatamer. This 3% redundancy yields terminally redundant and circularly permuted DNA^17,22^. The sequencing analysis of the ABMM1 genome revealed the presence of additional gene copy at both predicted termini (*g1*), providing evidence for terminal redundancy and confirming its circularly permuted genome structure through restriction enzyme analysis.

ABMM1 integrated its double-stranded DNA into the bacterial host genome as a prophage. This integration into the bacterial genome means that ABMM1 could persist in the host cell and be passed on to daughter cells, potentially providing a selective advantage or disadvantage to the host depending on environmental stressors or other factors. This study demonstrates the production of phage-resistant bacteria following the integration of ABMM1 into the host genome. To explore how bacteria develop resistance to phages, ABMM1 was co-cultured with the host for an extended period, and phage-resistant strains were subsequently isolated. Of the four strains that were analyzed, three exhibited the prophage form of ABMM1 in their genomes, as seen by the presence of a band in the PCR experiment using phage-specific primers (Gp48 and Gp73) encoding integrase and phage tail tip lysozyme of ABMM1, respectively (Fig. 5a and Supplementary Fig. S4) and an additional band in PFGE analysis (Fig. 5b) when compared to the wild-type strain, except R4. It was speculated that the resistance of R4 to ABMM1 might be due to "phenotype resistance" or the adaptation of phage tolerance^26^, leading to the loss of resistance to phage after several subcultures without phage stress. Another speculation is that R4 has mutated the phage receptor, it is sometimes possible that the receptor is under phage variation, which would explain the reversion of the phage sensitivity. Phages possess various characteristics that make them suitable candidates for therapeutic interventions. Notably, phages chosen for therapeutic purposes ought to have demonstrated the capability to effectively eliminate target bacteria through efficient bactericidal activity and induce complete eradication of infection. Additionally, the phage should not have the propensity to transduce host bacteria and should demonstrate the desired host range. Lastly, screening for toxin genes is crucial to identifying and eliminating any potential risks associated with phage therapy^27^. As precautionary measures, phages containing virulence and antibiotic-resistance genes should not be employed as biocontrol agents. Although ABMM1 will integrate into the bacterial genome under long-term co-culture with the host, the genomic analysis revealed that it has no virulence or antibiotic-resistance genes. These findings suggest that ABMM1 can be utilized safely as a therapeutic agent.

Using temperate phages as therapy is not without its challenges. Temperate phages can transfer genetic material between bacterial hosts, contributing to bacterial evolution and the acquisition of new traits^28^. We also found that somehow temperate phages have a narrower host range compared to lytic phages, as proven by our previous study about lytic phage that has a broader host range compared to ABMM1^7^. Although usually avoided, the use of temperate phages as a form of therapy has been explored in some studies. For example, in one study, the use of *Pseudomonas* phage D3112 resulted in a significant reduction in mortality in *Drosophila* infected with PA14 due to its ability to impair the twitching motility of the bacteria^29^. Meader et al.^30^ also employed a temperate phage to control *Clostridium difficile*. In our study, it was found that even after ABMM1 was co-cultured with the host for a period of time, phage-resistant mutants would appear due to the integration of the ABMM1 genome into the host chromosome, but as long as ABMM1 with a high MOI was used, both in vitro and in vivo experiments could significantly reduce bacterial growth and rescue zebrafish that die from bacterial infection. Thus, this study is the first to demonstrate the efficacy of temperate phage as an antimicrobial therapy in a zebrafish model infected with *A. baumannii*, despite previous studies using temperate phage W5 to infect *A. baumannii* strain Ab1 in vitro^31^. To ensure safety, the concern of phage integration into the host genome can be mitigated by employing phage-derived proteins, such as ABMM1 lysin, as potent bacteriolytic agents or by engineering a derivative of ABMM1 lacking the integrase gene block^32,33^. Lastly, a recent study has demonstrated the synergistic effect of *A. baumannii* phage and natural compounds, such as essential oils and plant extracts^34^. However, further research is necessary to optimize this approach and overcome the limitation of using temperate phage alone.

## Materials and methods

### Bacterial strains, media, and growth conditions

The bacteria in this study were clinical isolates of *A. baumannii* obtained from Taipei Veterans General Hospital (TVGH) in Taiwan, and reference strains *A. baumannii* ATCC 17978 and 19606. All strains were cultured in Lysogeny Broth (LB) and LB agar (Miller) at 37 °C with shaking at 150 rpm. *A. baumannii* growth was monitored turbidimetrically by measuring optical density at 600 nm (OD_600_), in which an OD unit of 1.0 corresponded to around 3 × 10^8^ CFU/ml.

### Phage isolation and purification

Phage was isolated from wastewater collected around Buddhist Tzu Chi Hospital using previously reported methods^7^. First, phage was amplified and high phage titer (100 ml containing ~ 10^11^ PFU/ml) was concentrated by centrifugation (18,000 rpm; Beckman Coulter Avanti JXN-26 centrifuge, JA-25.50 rotor; Beckman Coulter, Brea, CA, USA) for 2 h at 4 °C. The pellets were suspended in SM buffer (0.05 M Tris–HCl, pH 7.5 containing 0.1 M NaCl, 0.008 M MgSO4·7H_2_O, and 0.01% gelatin) and purified by banding on the step gradient of CsCl (ρ = 1.43, 1.45, and 1.5 g/cm^3^), followed by ultracentrifugation at 30,000 rpm (Beckman Coulter Optima XPN-100 ultracentrifuge, SW 41 Ti rotor; Beckman Coulter, Brea, CA, USA) for 3 h, at 4 °C. The banded phage particles were recovered and desalted by dialysis.

### Transmission electron microscopy

Transmission Electron Microscopy (TEM) examined phage morphology as previously described^7^. Briefly, a drop of 10^10^ PFU/ml of dialyzed phage particles was applied to the surface of a formvar-coated grid (300 mesh copper grids) (EMS, Hatfield, PA, USA), negatively stained with 2% uranyl-acetate and then examined in Hitachi H-7500 TEM operated at 80 kV.

### Phage host range analysis

According to a previous study, the host range of the phage was tested against 38 clinical isolates of *A. baumannii* from TVHG and 2 ATCC strains by spot test^35^. Briefly, *A. baumannii strains* were grown overnight in LB broth and 200 µl of strain culture was mixed with melted 0.7% LB agar and overlaid onto LB agar plate, 5 µl of phage lysate (10^6^ PFU/ml) was spotted onto bacteria lawn and incubated overnight at 37 °C. A clear zone indicated the sensitivity of bacteria to the phage.

### Phage adsorption analysis

Phage adsorption analysis was done according to previous protocol described^9^. Briefly, bacterial cells were infected with phage to give an MOI of 0.0005 and incubated at 37 °C with shaking. Samples (100 µl) were taken at 0, 2, 4, 6, 8, 10, 15, 20, and 30 min, and suspended in 0.9 ml of cold LB medium, followed by centrifugation at 12,000 rpm for 5 min (Heraeus Biofuge Pico microliter centrifuge, #3325 rotor; Heraeus instrument, Hanau, Germany) at room temperature. Samples were plated using the double-layer method to determine unabsorbed phage titer. The phage adsorption efficiency was calculated with the equation (initial phage titer − unabsorbed phage titer in the supernatant)/initial phage titer multiplied by 100.

### Phage one-step growth analysis

Bacterial Cells were infected with phage at MOI of 0.0001 and allowed to adsorb for 15 min on ice. The mixture was centrifuged (12,000 rpm, 10 min) with Heraeus Biofuge Pico microliter centrifuge, #3325 rotor and the pellets containing infected cells were suspended in 20 ml of LB, followed by incubation at 37 °C. Samples were collected at 10-min intervals up to 80 min, and immediately diluted in LB medium, then plated using the double-layer method to determine the phage titer. The latent period and burst size were determined as described previously^36^.

### Bacterial growth reduction test

To check the bacteriolytic activity of phage, exponential growth-phase cultures of host cells were infected with phage at MOI of 0.001, 0.01, 0.1, or 1 at 37 °C for 5 h, and the OD_600_ was measured every hour using a cell density meter (Ultrospec 10, Biosciences, Amersham, United Kingdom). The experiments were performed thrice, and the value was converted to CFU/ml by calculating colonies on dilution plating.

### Phage DNA isolation

Phage suspension was treated with 1 µg/ml DNase I and 10 µg/ml RNase A (Promega, Madison, WI, USA) for 3 h. After that, the phage particles were concentrated with 20% PEG 6000 and 2.5 M NaCl followed by centrifugation at 18,000 rpm (Beckman Coulter Avanti JXN-26 centrifuge, JA-25.50 rotor; Beckman Coulter, Brea, CA, USA) for 2 h at 4 °C. Finally, the concentrated phage encountered extraction with phenol/chloroform and phage DNA was precipitated by ethanol^7^.

### Pulse-field gel electrophoresis (PFGE)

PFGE was done for phage DNA size determination and was performed as described previously using a CHEF-DR III System (Bio-Rad Laboratories, Hercules, CA, USA) at 6 V/cm with pulse ramps from 1 to 6 s for 20 h for undigested phage DNA at 14 °C in 0.5 × Tris–borate-EDTA buffer (TBE)^7^. A Midrange PFG Marker (New England BioLabs, Ipswich, MA, USA) was used as the molecular size standard.

For determining the integration of phage DNA into the bacterial genome, PFGE of TV2199 WT and phage-resistant strains were done according to a previous study with some modifications^37^. Briefly, fresh bacterial colonies were suspended in cell suspension buffer (100 mM Tris, 100 mM EDTA, pH 8.0), mixed with the same 1.6% melting agarose volume, and distributed in plug molds. Genomic DNA in the agarose plugs was lysed in the cell lysis buffer (50 mM Tris, 50 mM EDTA, pH 8.0 + 1% sarcosine) overnight, washed, and digested with *Apa*I restriction enzyme (Fermentas, Waltham, MA, USA). A lambda PFG Ladder (New England BioLabs) was used as a molecular size marker. The electrophoresis was performed using the same instrument as above at 6 V/cm with pulse ramps from 5 to 20 s for 23 h at 14 °C in 0.5 × TBE.

### Analysis of phage structural proteins

Sodium dodecyl-sulfate polyacrylamide gel electrophoresis (SDS-PAGE) was performed according to the previous protocol^38^. Briefly, phage particles (2 × 10^10^ PFU/ml) were mixed with sample buffer (1 M Tris–HCl pH 6.8 containing 10% sodium dodecyl sulfate, 20% glycerol, 1 M dithiothreitol, and 0.02% bromophenol blue) and heated in boiling water for 10 min, followed by separation of the protein in SDS–polyacrylamide gel (12%) electrophoresis. Protein bands were detected by Coomassie blue staining. The bands were cut out of the gel and digested with trypsin, and the identification was carried out by LC–MS/MS analysis. The raw data were searched against a local database of all possible peptide spectra deduced from the ABMM1 genome sequence.

### Thermal and pH stability test

The stability of phage in particular thermal and pH was done according to the protocol described previously with modification^7^. Briefly, the phage suspension was incubated at 4, 22, 37, 50, 60, and 70 °C for 1 h; and for pH stability, the phage was incubated at pH 3, 5, 7, 9, and 11 for 1 h at 37 °C. The phage titer from collected samples was determined by using a double-layer method. All experiments were performed three times.

### DNA sequencing and genome analysis

The Phage DNA genome was sequenced by Allbio Company (Taipei, Taiwan). Genomic DNA was fragmented to an average size of 300–350 bp that were then processed to generate DNA libraries. The qualified libraries were sequenced pair end PE150 on the Illumina HiseqXten/Novaseq/MGI2000 system. The reads were assembled using velvet or Novoplasty, gapfilled with SSPACE and Gapfiller^39–43^. The lifecycle of phage was predicted online by PhageAI^44^. Phage whole genome was subjected to BLASTN (megablast) to find highly similar phage genome sequences in the NCBI database^45^. Potential genes was identified by RAST and was checked manually^46^. BLASTP was used to predict function to the annotated gene products against non-redundant (nr) protein sequences, protein data bank (PDB), and HHpred^47,48^. Phage annotation map was generated by SnapGene^®^ Viewer v.6.1.2 (http://www.snapgene.com/). Antibiotic resistance genes and virulence factors were detected by ResFinder and VirulanceFinder^49^. The presence of tRNAs in the genome was searched using tRNAscan-SE and ARAGORN^50,51^. Prophage Hunter was used to identify prophage in *A. baumannii* genomes^52^. Comparative genome analysis was conducted using Mauve alignment and Easyfig 2.2.5^53,54^.

### Phage genome termini analysis in vitro

The phage DNA genome was used as a template for Sanger-based sequencing using specific forward and reverse primers designed to reach the putative genome 5′ and 3′ termini (Supplementary Table S5). Later, phage DNA (200 ng) was digested using *Ava*I, *Xho*I, *Bgl*I, and *Nhe*I (New England BioLabs) that resulted several well separated bands when based on in silico. The digestion were carried out in the final of 15 µl and were incubated for 16 h at 37 °C. To check if the phage genome having cohesive ends, *Ava*I digested phage DNA was analyzed in agarose gel after heat treatment at 80 °C for 15 min and either cooled rapidly on ice or allowed to cool slowly at room temperature.

### Construction of phylogenetic tree

To find the phage's closest relative and identify phage-family level classification, the whole genome sequence was used as input in VIPtree server version 3.6 against all reference phages in the database and formed phylogenetic tree^55,56^. The amino sequence of phage TerL subunit and was aligned with those of the closely related phages and int was aligned with different *A. baumannii* integrases using MUSCLE. The tree were constructed using the neighbor-joining method with 1000 bootstrap replication in MEGA X^57^.

### Isolation of phage-resistant strains and determination of phage lysogenic activity

To test the ability of phage to integrate into host DNA and form prophage, phage-resistant strains were isolated according to a previous study with some modifications^18^. TV2199 overnight culture was spread on LB agar plate and allowed to dry, and phage stock (10^10^ PFU/ml) was dropped onto the bacterial lawn and incubated for 48 h at 37 °C. Surviving colonies that grow on the clear plaques were collected and tested for phage resistance by spot test. To confirm the presence of phage DNA in the bacterial genome, phage-resistant strains were used as templates in phage-specific PCR using primers listed in Supplementary Table S5. PCR was conducted at 96 °C for 3 min, 30 cycles of denaturation (96 °C, 1 min), annealing (59 °C, 1 min) and extension (72 °C, 1 min), and a final extension step at 72 °C for 5 min. PCR products were analyzed by agarose gel electrophoresis, followed by ethidium bromide staining and visualization under a UV trans-illuminator.

### The effect of phage treatment by a zebrafish model of bacterial infection

The zebrafish (*Dario rerio*) lines used in this study were the wild-type AB variety. Mixed male and female zebrafish populations were kept in 9 L tanks at 28 °C and maintained in 14 h light/10 h dark cycle. The median lethal dose (LD_50_) was first determined by injecting a different dose of *A. baumannii* TV2199 (10^5^, 10^6^, and 10^7^ CFU) through the zebrafish cloaca using the insulin needle after being anesthetized with 160 mg/ml tricaine. The survival rate was monitored by observation for 24 h. To see the phage therapy effect on bacterial infection in zebrafish model, the adult disease-free zebrafish were divided into four groups (n = 8). The three groups were injected with LD_50_ of *A. baumannii* TV2199*.* After 30 min, different doses of ABMM1 (MOI 1 and 10) were injected into the second and third group, respectively. The fourth group was zebrafish injected with only phage to check its safety to use as therapy. The survival rate was assessed every 24 h until 72 h.

Zebrafish use was performed in agreement with ARRIVE guidelines (https://arriveguidelines.org). All methods were performed in accordance with the relevant guidelines and regulations (Institutional Animal Care and Use Committee of Tzu Chi University) (IACUC approval no. 109018).

### Statistical analysis

Data were shown as mean ± SEM. Results were compared by one-way analysis of variance (ANOVA) followed by Dunnett’s post-test. Survival analysis was interpreted by Mantel–Cox test. Statistical analysis was performed by GraphPad Prism version 9.4.0.

### Genome availability

The genome sequence of phage ABMM1 has been deposited in GenBank under the accession number OQ471969.

### Institutional review board statement

Zebrafish use was performed in agreement with ARRIVE guidelines (https://arriveguidelines.org). The laboratory protocol was approved by the Institutional Animal Care and Use Committee of Tzu Chi University (IACUC Approval No.: 109018).

## Supplementary Information

Supplementary Information.

## Data Availability

The whole genome sequencing of ABMM1 has been done in the study and has been deposited in NCBI GenBank through Bankit under accession number OQ471969. The data presented in this study are available in this article and supplementary material here.
