# Supplementary material for: Characterization of a novel and active temperate phage vB_AbaM_ABMM1 with antibacterial activity against Acinetobacter baumannii infection
Source: Sci Rep. 2023 Jul 13;13:11347. doi: 10.1038/s41598-023-38453-7 (PMC10345192; doi:10.1038/s41598-023-38453-7)
Supplement: Supplementary file 1 — Supplementary Information. [file 41598_2023_38453_MOESM1_ESM.pdf]

Characterization of a novel and active temperate phage vB_AbaM_ABMM1 with antibacterial activity against *Acinetobacter baumannii* infection

Meity Mardiana [1], Soon-Hian Teh [2], Yun-Chan Tsai[3], Hsueh-Hui Yang[4], Ling-Chun Lin [5]* and Nien-Tsung Lin [1,5,]*

[1] Institute of Medical Sciences, Tzu Chi University, No. 701, Sec. 3, Zhongyang Rd., 97004 Hualien, Taiwan; meitymardiana@gmail.com (M.M.); ntlin@gms.tcu.edu.tw (N.-T.L)
[2] Division of Infectious Diseases, Department of Internal Medicine, Hualien Tzu Chi Hospital, Buddhist Tzu Chi Medical Foundation, No. 707, Sec. 3, Zhongyang Rd., 97004 Hualien, Taiwan; jimmyteh2000@gmail.com (S.-H.T)
[3] Department of Life Sciences, Tzu Chi University, No. 701, Sec. 3, Zhongyang Rd., 97004 Hualien, Taiwan; jim.tsai201248@gmail.com (Y.-C.T)
[4] Department of Medical Research, Hualien Tzu Chi Hospital, Buddhist Tzu Chi Medical Foundation, No. 707, Sec. 3, Zhongyang Rd., 97004 Hualien, Taiwan; hhyang@tzuchi.com.tw (H.-H.Y)
[5] Master Program in Biomedical Sciences, School of Medicine, Tzu Chi University, No. 701, Sec. 3, Zhongyang Rd., 97004 Hualien, Taiwan; lcl2108@gmail.com (L.-C.L)

* Correspondence: lcl2108@gmail.com (L.-C.L); ntlin@gms.tcu.edu.tw (N.-T.L); Tel.: +886-3-8565301 #2108 (L.-C.L), #2080 (N.-T.L); Fax: +886-3-8566724 (N.-T.L)

Supplementary Table S1. Host range analysis of ABMM1

Strain	Infectivity	Source
TV311	+	TVGH[a]
TV338	+	TVGH[a]
TV479	++	TVGH[a]
TV481	-	TVGH[a]
TV485	-	TVGH[a]
TV514	-	TVGH[a]
TV530	-	TVGH[a]
TV574	-	TVGH[a]
TV612	+	TVGH[a]
TV618	-	TVGH[a]
TV619	-	TVGH[a]
TV962	-	TVGH[a]
TV967	-	TVGH[a]
TV1003	-	TVGH[a]
TV1093	-	TVGH[a]
TV1544	-	TVGH[a]
TV1731	-	TVGH[a]
TV1733	+	TVGH[a]
TV1734	+	TVGH[a]
TV1735	-	TVGH[a]
TV2050	-	TVGH[a]
TV2088	+	TVGH[a]
TV2177	++	TVGH[a]
TV2199	++	TVGH[a]
TV2606	+	TVGH[a]
TV2629	++	TVGH[a]
TV2655	-	TVGH[a]
TV2701	-	TVGH[a]
TV2704	+	TVGH[a]
TV2716	-	TVGH[a]
TV2718	-	TVGH[a]
TV2722	+	TVGH[a]
TV2729	-	TVGH[a]
TV2738	-	TVGH[a]
TV2747	+	TVGH[a]

TV2749	+	TVGH[a]
TV2755	-	TVGH[a]
TV2784	+	TVGH[a]
ATCC 17978	-	ATCC[b]
ATCC 19686	-	ATCC[b]

[a]Taipei Veteran General Hospital

[b]American Type Culture Collection

(++): clear zone, (+): turbid, (-): no lysis (absence of clear zone).

Supplementary Table S2. G+C content comparison of ABMM1 and some *A. baumannii* whole genomes

Strain	G + C content (%)	Accession number
ABMM1	37.3	OQ471969
DT-Ab003	39.1	CP050916
DT-Ab020	39.1	CP050911
DD520	39.1	CP075321
DT-Ab022	39.2	CP050907
DT-Ab057	39.1	CP050904

Supplementary Table S3. Genome annotations of ABMM1

ORF	Start	End	Strand	Predicted protein function	AA	MW (kDa)	BLASTP e-value (% Identitiy)	Hhpred e-value (%probability)	Best hit
	1	127	+	repeat region					
1	1(75542)	138(75730)	+	TraR/Dksa C4-type zinc finger protein	45	5.36	2E-51 (97)		
2	150	662	+	hypothetical protein	170	19	2E-51 (97)		QHJ78693.1
3	682	2673	+	terminase, large subunit	663	75.43		8.5e-29 (99.97)	3CPE_A
4	2746	4284	+	tail-collar fiber protein	512	53.62		9e-2 (97.29)	PF12571.11
5	4299	4577	+	baseplate wedge subunit	92	10.85		8.6e-9 (98.83)	5HX2_F
6	4579	5709	+	tail protein	376	43.4		9.2e-2 (97.29)	PF12571.11
7	5699	6247	+	hypothetical protein	182	20.74	9E-128 (98.35)		WP_174737935.1
8	6244	6546	+	hypothetical protein	100	11.1	2E-25 (50.52)		QHJ83363.1
9	6701	8479	+	Head vertex protein	592	63.65		6.8e-36 (100)	1YUE_A
10	8543	9166	+	hypothetical protein	207	22.16	2E-145 (100)		WP_000718475.1
11	9171	9458	+	hypothetical protein	95	10.22	2E-60 (100)		WP_000151780.1
12	9575	10870	+	tail sheath protein	431	47.54			3J2M_V
13	10889	11656	+	hypothetical protein	255	27.86	0 (100)		WP_033917782.1
14	11718	11996	+	tail sheath protein	92	10.32		2e-29 (100)	3J2M_V
15	12978	12082	-	phage antirepressor N-terminal domain-containing protein	298	34.5	0 (100)		WP_000210194.1
16	13185	13436	+	hypothetical protein	83	9.57	2E-54 (100)		WP_000229653.1
17	13728	13603	-	phage antirepressor N-terminal domain-containing protein	41	4.74	1e-19 (100)		WP_254977142.1
18	13970	14257	+	prevent host death protein	95	10.81		4.2e-7 (98.73)	3HS2_H
19	14220	14486	+	mRNA interferase RelE	88	10.35		9.4e-15 (99.7)	4FXI_C
20	14613	15257	+	Tail tube protein	214	23.33		4e-10 (99.28)	5IV5_IA
21	15257	15706	+	hypothetical protein	149	16.56	1E-104 (100)		WP_001240941.1

ORF	Start	End	Strand	Predicted protein function	AA	MW (kDa)	BLASTP e-value (% Identitiy)	Hhpred e-value (%probability)	Best hit
22	15706	16599	+	hypothetical protein	297	32.28	0 (100)		WP_001240941.1
23	16609	26964	+	hypothetical protein	3451	372.61	0 (98.33)		WP_108584107.1
24	27076	27363	+	hypothetical protein	95	10.54	3E-41 (68.42)		AEQ18753.1
25	27363	28133	+	hypothetical protein	256	28.75	3E-153 (80.47)		YP_009813092.1
26	28355	28161	-	hypothetical protein	64	7.18	2E-38 (100)		WP_000852568.1
27	28514	30049	+	hypothetical protein	511	57.52	0 (100)		WP_000098517.1
28	30049	30702	+	hypothetical protein	217	24.21	9E-158 (100)		WP_000863706.1
39	30776	32728	+	hypothetical protein	650	73.6	0 (100)		WP_002093906.1
30	32728	34179	+	hypothetical protein	483	53.36	0 (100)		WP_000209673.1
31	34330	34959	+	hypothetical protein	209	23.74	4E-150 (100)		WP_000951724.1
32	34956	36035	+	XRE family transcriptional regulator	359	41.07	0 (100)		WP_000067934.1
33	36266	36964	+	hypothetical protein	232	27.5	1E-171 (100)		WP_000373973.1
34	38022	37123	-	phage antirepressor N-terminal domain-containing protein	299	34.91	0 (100)		WP_000067916.1
35	38224	38472	+	REPRESSOR	82	9.29		9.2e-4 (97.72)	1ADR_A
36	38560	39354	+	Baseplate tail-tube protein	264	28.65		2.4e-2 (96.96)	5IV5_r
37	39354	40052	+	hypothetical protein	232	26.94	1E-171 (100)		WP_000627455.1
38	40084	40263	+	hypothetical protein	59	6.83	4E-35 (100)		WP_000887468.1
39	40385	40672	+	Holing	95	11.03		8.9e-20 (99.84)	PF16082.8
40	40669	41439	+	putative lysin	256	28.61		4e-2 (97.15)	6IST_C
41	41936	41565	-	hypothetical protein	123	14.35	2e-84 (100)		MCL8351649.1
42	42048	42185	+	IS5/IS1182 family transposease	45	5.18	3e-25 (100)		TDH86709.1
43	43006	42308	-	hypothetical protein	232	27.26	73-170 (100)		WP_001072150.1
44	44458	43160	-	error-prone lession bypass DNA polymerase V	432	48.98	0 (62.35)		ASN69678.1

ORF	Start	End	Strand	Predicted protein function	AA	MW (kDa)	BLASTP e-value (% Identitiy)	Hhpred e-value (%probability)	Best hit
45	44956	44462	-	protein umuD	164	18.48	3e-67 (59.62)		ASN69677.1
46	45715	45071	-	hypothetical protein	214	25.55		8.4e-40 (100)	1ZN6_A
47	45829	46581	+	hypothetical protein	250	28.81	2e-180 (100)		WP_000171287.1
48	48099	46894	-	Integrase	401	46.79		4e-28 (99.97)	1Z1B_A
49	48745	48530	-	hypothetical protein	71	8.33	3e-46 (100)		WP_000362184.1
50	49469	48747	-	hypothetical protein	240	28.22	8e-177 (100)		WP_094914353.1
51	49945	49466	-	hypothetical protein	159	18.53	2e-18 (72.73)		UKM17098.1
52	50193	49948	-	hypothetical protein	81	9.9	8e-53 (100)		WP_005120240.1
53	51154	50195	-	hypothetical protein	319	35.87	0 (100)		WP_005120238.1
54	52272	51151	-	putative RecA family protein	373	41.21	0 (98.93)		AWD93183.1
55	52606	52283	-	hypothetical protein	107	12.63	2e-73 (100)		WP_000064472.1
56	53049	52609	-	hypothetical protein	146	15.92	3E-102 (100)		WP_148735590.1
57	53937	53266	-	Repressor	223	24.99		1.1e-19 (99.86)	3BDN_A
58	54065	54295	+	transcriptional repressor	76	8.56	3e-23 (48.48)		YP_009017575.1
59	54306	54626	+	hypothetical protein	106	11.83	4e-71 (100)		WP_000049332.1
60	54685	54975	+	hypothetical protein	96	11.27	4e-64 (100)		WP_104073961.1
61	54972	55886	+	DNA methyltransferase	304	33.52	0 (100)		WP_063963422.1
62	55883	56833	+	hypothetical protein	316	36.48	0 (100)		WP_104073943.1
63	56826	57575	+	hypothetical protein	249	28.35	0 (100)		WP_104073944.1
64	57572	57979	+	hypothetical protein	135	15.93	2e-91 (99.24)		CAP00675.1
65	57976	58260	+	hypothetical protein	94	10.63	3e-63 (100)		CAP00675.1
66	58337	59116	+	hypothetical protein	259	30.01	0 (100)		WP_000171353.1
67	59165	59380	+	hypothetical protein	71	8.24	2e-45 (100)		SST72184.1

ORF	Start	End	Strand	Predicted protein function	AA	MW (kDa)	BLASTP e-value (% Identitiy)	Hhpred e-value (%probability)	Best hit
68	59643	60341	+	RusA family crossover juction endodeoxyribonuclease	232	26.73	8e-171 (100)		WP_000592662.1
69	60342	61028	+	helix-turn-helix domain-containing protein	228	25.79	5e-165 (100)		WP_001085636.1
70	61042	61878	+	hypothetical protein	278	31.45	0 (99.64)		HAV2988592.1
71	61996	62418	+	hypothetical protein	140	15.73	7e-93 (98.57)		HAV2988591.1
72	62428	62730	+	hypothetical protein	100	11.38	4E-63 (100)		WP_249672623.1
73	62854	65142	+	phage tail tip lysozyme	762	82.66	0 (96.46)		WP_239930480.1
74	65156	66211	+	hypothetical protein	351	39.98	0 (100)		WP_000172885.1
75	66535	67476	+	Type II toxin-antotoxin system HicB family toxin	313	36.21	0 (100)		WP_000801068.1
76	67541	68440	+	phage antirepressor N-terminal domain-containing protein	299	35	0 (100)		WP_001259764.1
77	68928	68512	-	hypothetical protein	138	16	1e-94 (100)		WP_000274053.1
78	69126	70067	+	tetratricopeptide repeat protein	313	34.89	0 (100)		WP_000535154.1
79	70033	70623	+	hypothetical protein	196	22.7	1e-137 (100)		ASF76611.1
80	70623	70961	+	hypothetical protein	112	12.52	5e-76 (100)		WP_000633139.1
81	70942	72387	+	baseplate wedge protein	481	54.55		1.4e-39 (100)	5HX2_D
82	72375	72989	+	hypothetical protein	204	23.43	3e-150 (100)		WP_000371256.1
83	73068	73526	+	hypothetical protein	152	17.08	1e-106 (100)		WP_001183519.1
84	73538	73765	+	hypothetical protein	75	8.84	5e-45 (96)		WP_057694966.1
85	73838	74743	+	hypothetical protein	301	33.83	0 (100)		WP_000157185.1
86	74746	75549	+	hypothetical protein	267	30.15	0 (100)		WP_001108875.1
	75542	75731	+	repeat region					

Supplementary Table S4. Identified prophage in top 5 *A. baumannii* strains

Strain	Candidate ID	Sequence ID	Start	End	Length	Category	Score	Closest phage	Gene number
DT-Ab003	Candidate_1	CP050916.1	1530369	1546610	16242	Inactive	0.24	N/A	16
	Candidate_2	CP050916.1	1263419	1312467	49049	Ambiguous	0.55	*Acinetobacter* phage Ab105-1phi	64
	Candidate_3	CP050916.1	923955	956590	32636	Inactive	0.11	*Prochlorococcus* phage P-SSM2	32
	Candidate_4	CP050916.1	2103332	2131320	27989	Inactive	0.08	*Moraxella* phage Mcat3	23
	Candidate_5	CP050916.1	489237	521239	32003	Inactive	0.04	*Moraxella* phage Mcat16	30
	Candidate_6	CP050916.1	336865	374342	37478	Inactive	0.04	*Aeromonas* phage SW69-9	38
	Candidate_7	CP050916.1	2763970	2811793	47824	Active	0.94	*Acinetobacter* phage YMC11/11/R3177	70
	Candidate_8	CP050916.1	2963717	2994639	30923	Ambiguous	0.67	*Acinetobacter* phage Ab105-2phi	34
	Candidate_9	CP050916.1	3798986	3818917	19932	Ambiguous	0.5	*Moraxella* phage Mcat16	15
	Candidate_10	CP050916.1	2648638	2673661	25024	Inactive	0.08	N/A	20
	Candidate_11	CP050916.1	1717836	1735305	17470	Active	0.82	*Clostridium* phage CDSH1	17
	Candidate_12	CP050916.1	1306319	1325371	19053	Ambiguous	0.64	*Acinetobacter* phage Ab105-1phi	25
	Candidate_13	CP050916.1	2724084	2758249	34166	Active	0.92	*Acinetobacter* phage Bphi-B1251	50
	Candidate_14	CP050916.1	3022008	3060942	38935	Active	0.86	*Acinetobacter* phage Ab105-1phi	52
	Candidate_15	CP050916.1	433648	463276	29629	Inactive	0.37	*Bacillus* phage vB_BpuM-BpSp	42
DT-Ab020	Candidate_1	CP050911.1	489273	521275	32003	Inactive	0.04	*Moraxella* phage Mcat16	30
	Candidate_2	CP050911.1	2154566	2182554	27989	Inactive	0.08	*Moraxella* phage Mcat3	23
	Candidate_3	CP050911.1	923991	956626	32636	Inactive	0.11	*Prochlorococcus* phage P-SSM2	32
	Candidate_4	CP050911.1	1178644	1230371	51728	Active	0.99	*Acinetobacter* phage Ab105-1phi	68
	Candidate_5	CP050911.1	2699872	2724895	25024	Inactive	0.08	N/A	20
	Candidate_6	CP050911.1	433684	463312	29629	Inactive	0.37	*Bacillus* phage vB_BpuM-BpSp	42
	Candidate_7	CP050911.1	3073242	3112176	38935	Active	0.86	*Acinetobacter* phage Ab105-1phi	52
	Candidate_8	CP050911.1	1316915	1380528	63614	Inactive	0.49	*Acinetobacter* phage Ab105-1phi	84
	Candidate_9	CP050911.1	336901	374378	37478	Inactive	0.04	*Aeromonas* phage SW69-9	38
	Candidate_10	CP050911.1	3005540	3030254	24715	Ambiguous	0.68	*Acinetobacter* phage Ab105-2phi	24
	Candidate_11	CP050911.1	2762822	2818270	55449	Active	0.81	*Acinetobacter* phage Bphi-B1251	71
	Candidate_12	CP050911.1	2801401	2868521	67121	Active	0.91	*Acinetobacter* phage YMC11/11/R3177	83
DD520	Candidate_1	CP075321.1	1761801	1776826	15026	Ambiguous	0.62	*Clostridium* phage CDSH1	13
	Candidate_2	CP075321.1	714040	770426	56387	Ambiguous	0.65	*Acinetobacter* phage Ab105-1phi	74
	Candidate_3	CP075321.1	1415937	1452787	36851	Inactive	0.02	*Streptococcus* phage phi1207.3	33
	Candidate_4	CP075321.1	334057	371535	37479	Inactive	0.04	*Aeromonas* phage SW69-9	38
	Candidate_5	CP075321.1	3004771	3021682	16912	Active	0.87	*Acinetobacter* phage Ab105-1phi	25

Strain	Candidate ID	Sequence ID	Start	End	Length	Category	Score	Closest phage	Gene number
	Candidate_6	CP075321.1	2760073	2832335	72263	Active	0.81	*Acinetobacter* phage YMC11/11/R3177	89
	Candidate_7	CP075321.1	1039525	1101078	61554	Ambiguous	0.8	*Acinetobacter* phage Bphi-B1251	72
	Candidate_8	CP075321.1	2693142	2718165	25024	Inactive	0.08	N/A	20
	Candidate_9	CP075321.1	963617	996252	32636	Inactive	0.11	*Prochlorococcus* phage P-SSM2	32
DT-Ab022	Candidate_1	CP050907.1	923964	956599	32636	Inactive	0.11	*Prochlorococcus* phage P-SSM2	32
	Candidate_2	CP050907.1	1178617	1230344	51728	Active	0.99	*Acinetobacter* phage Ab105-1phi	68
	Candidate_3	CP050907.1	2774606	2808771	34166	Active	0.93	*Acinetobacter* phage Bphi-B1251	51
	Candidate_4	CP050907.1	1316888	1380501	63614	Inactive	0.49	*Acinetobacter* phage Ab105-1phi	84
	Candidate_5	CP050907.1	336874	374351	37478	Inactive	0.04	*Aeromonas* phage SW69-9	38
	Candidate_6	CP050907.1	2699160	2724183	25024	Inactive	0.08	N/A	20
	Candidate_7	CP050907.1	433657	463285	29629	Inactive	0.37	*Bacillus* phage vB_BpuM-BpSp	42
	Candidate_8	CP050907.1	489246	521248	32003	Inactive	0.04	*Moraxella* phage Mcat16	30
	Candidate_9	CP050907.1	3049159	3093844	44686	Inactive	0.46	*Acinetobacter* phage Ab105-1phi	56
	Candidate_10	CP050907.1	2814492	2862315	47824	Active	0.93	*Acinetobacter* phage YMC11/11/R3177	70
DT-057	Candidate_1	CP050904.1	1756685	1771710	15026	Ambiguous	0.62	*Clostridium* phage CDSH1	13
	Candidate_2	CP050904.1	336866	374343	37478	Inactive	0.04	*Aeromonas* phage SW69-9	38
	Candidate_3	CP050904.1	489238	521241	32004	Inactive	0.04	*Moraxella* phage Mcat16	30
	Candidate_4	CP050904.1	1179800	1231528	51729	Active	0.99	*Acinetobacter* phage Ab105-1phi	68
	Candidate_5	CP050904.1	433649	463277	29629	Inactive	0.37	*Bacillus* phage vB_BpuM-BpSp	42
	Candidate_6	CP050904.1	3005601	3043016	37416	Ambiguous	0.76	*Acinetobacter* phage Ab105-1phi	51
	Candidate_7	CP050904.1	2657886	2682909	25024	Inactive	0.08	N/A	20
	Candidate_8	CP050904.1	2143391	2171379	27989	Inactive	0.08	*Moraxella* phage Mcat3	23
	Candidate_9	CP050904.1	2773218	2821041	47824	Active	0.93	*Acinetobacter* phage YMC11/11/R3177	70
	Candidate_10	CP050904.1	925147	957782	32636	Inactive	0.11	*Prochlorococcus* phage P-SSM2	32
	Candidate_11	CP050904.1	2720836	2776284	55449	Active	0.82	*Acinetobacter* phage Bphi-B1251	72
	Candidate_12	CP050904.1	1318072	1381685	63614	Inactive	0.49	*Acinetobacter* phage Ab105-1phi	84
	Candidate_13	CP050904.1	3952221	3985219	32999	Inactive	0.02	*Moraxella* phage Mcat16	28

A score closer to 1 indicates a higher probability of the prophage being active (putative prophage region scoring >0.8 is active, 0.5–0.8 as ambiguous, and <0.5 is inactive).

Supplementary Table S5. PCR primers used in this study

Primer Name	Primer Sequence	Product Size (bp)	Reference
Gp48-ABMM1_F	5'-ATGGCTTTAACTGAAGTGTGGCTGAAAGC-3'	1206	This study
Gp48-ABMM1_R	5'-GCTTAAATATTAGTCAGAGAGTTAAGTTTCTTCAACC-3'		
Gp73-ABMM1_F	5’-GGATCCATGACAGATTCAAATCACAATAACC-3’	2289	This study
Gp73-ABMM1_R	5’-CTCGAGCTAAACGTCTAATTTCCCCATTC-3’		
Ab003-F	5'-CCACCATTCGGGAATTTCTTTTAATGGTGC-3'	1639	This study
Ab003-R	5'-GTGCTTGGTATATGCCTGAGACCCAATTAAC-3'		

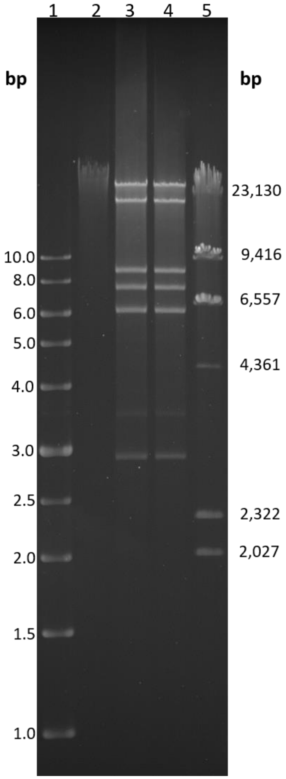

Supplementary Figure S1. Verification of cohesive ends in the ABMM1 genome

Lane 1, 1-Kb DNA ladder and lane 5, Lambda/*Hind*III ladder (New England Biolabs) Lane 2, undigested ABMM1 DNA. *Ava*I-digested phage DNA, heated after digestion at 80 °C for 15 min, followed by rapid cooling on ice (lane 2) or at room temperature (lane 3). No significant pattern difference between the two condition. The original gel is presented in Supplementary Fig. S13

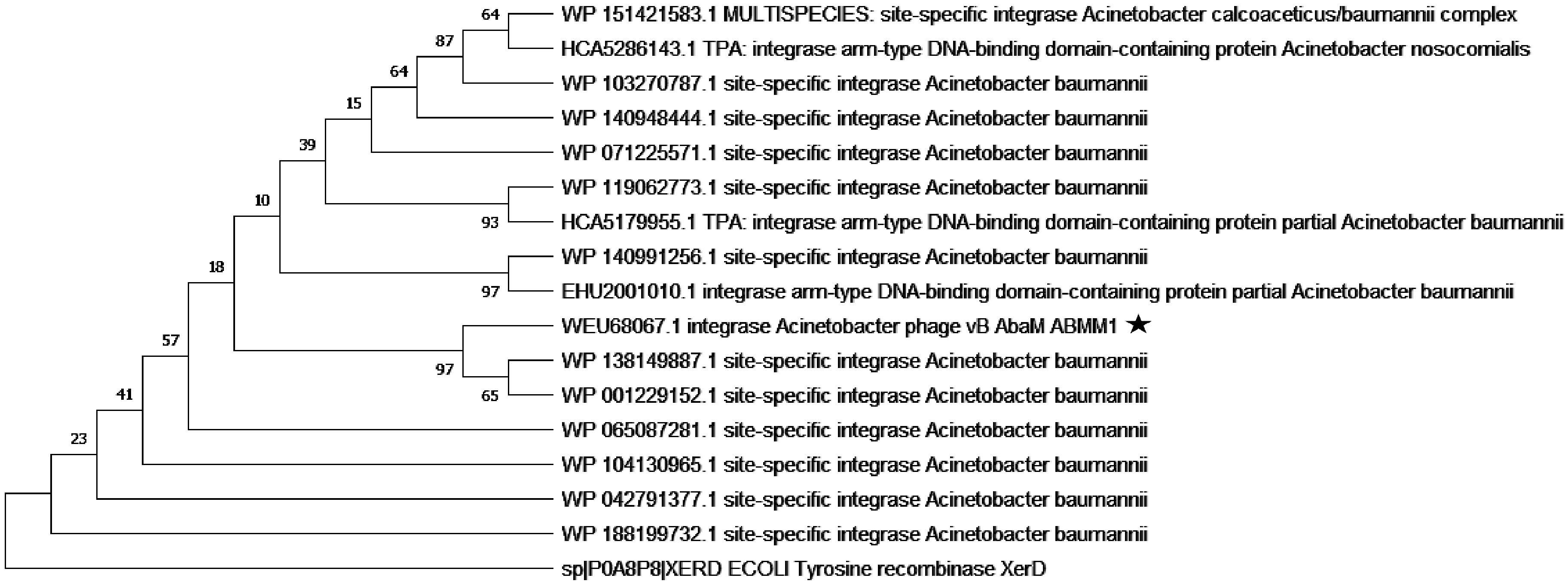

Supplementary Figure S2. The phylogenetic tree of ABMM1 integrase with different site-specific *Acinetobacter* integrases

ABMM1 belongs to *XerC* superfamily. The tree was constructed using MEGA X neighbor-joining analysis method with bootstrapping set to 1000 tree branches are proportional to branch lengths.

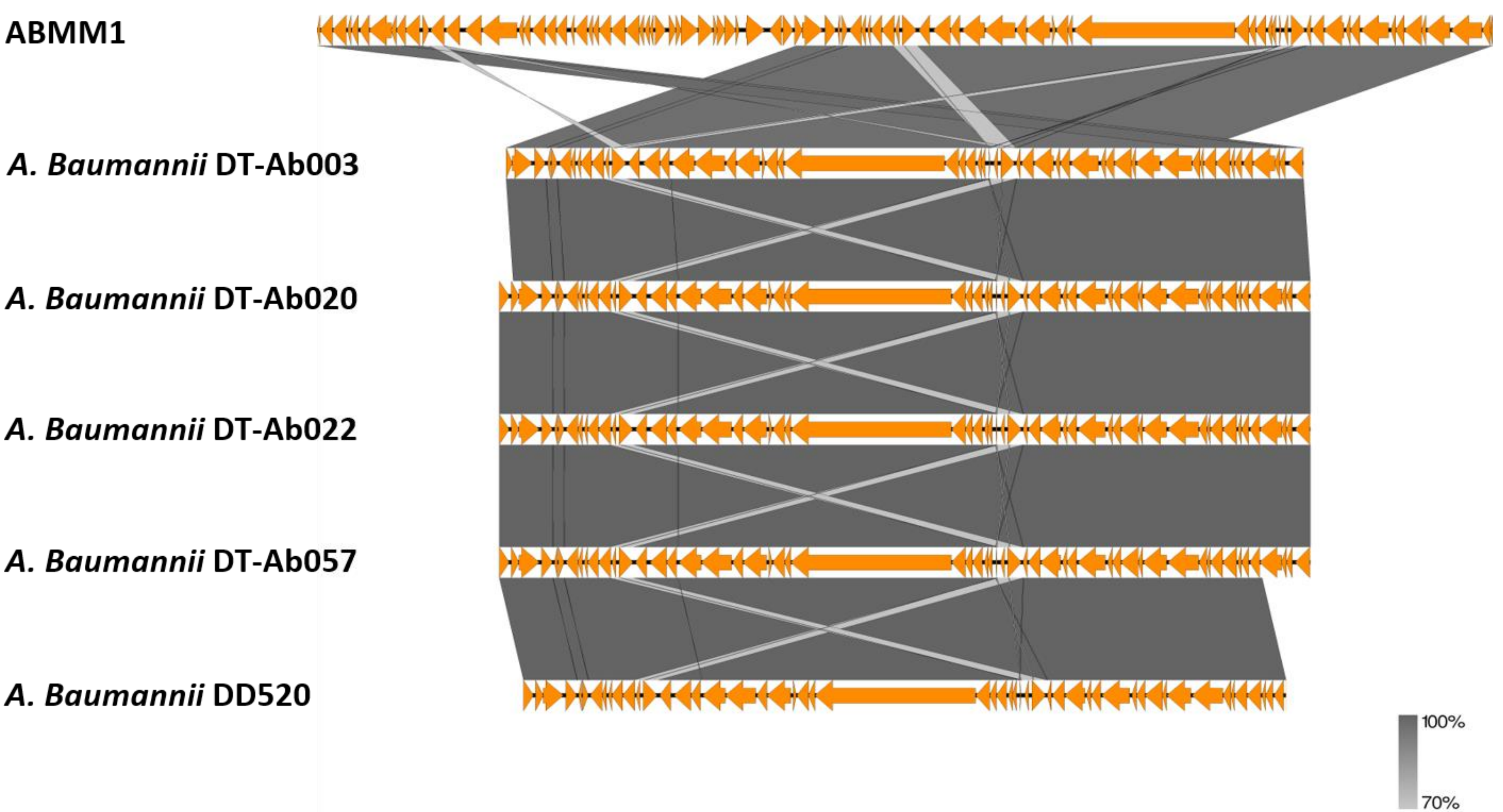

Supplementary Figure S3. The deletion and rearrangement of truncated prophage were observed within a specific bacterial genome region that exhibited homology with the genome.

Blastn alignment of ABMM1 between the two direct repeats at the end and corresponding regions in the *A. baumannii* strain DT-Ab003 (CP050916) (nt 2,977,165 – 3,045,803); DT-Ab020 (CP050911) (nt 3,027,508 – 3,097,037); DT-Ab022 (CP050907) (nt 3,002,032 – 3,077,019); DT-Ab057 (CP050904) (nt 2,960,758 – 3,035745); and DD520 (CP075321) (nt 2,959,037 – 3,008,168) was performed by Easyfig. Blue-coloured box represents phages genes that were lost in the truncated prophage. ABMM1 genome arrangement was reversed to match the organization of prophage genomes. The gradient scale indicates the similarity range.

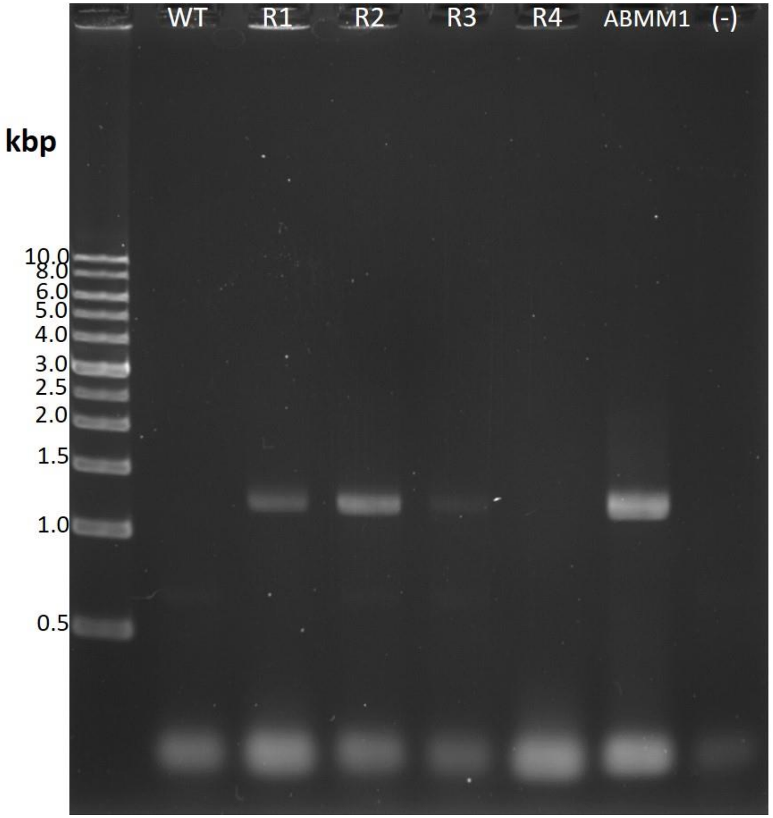

Supplementary Figure S4. PCR amplification of integrase protein gene of ABMM1 from TV2199 WT and phage-resistant strains

PCR amplified target band of 1,206 bp, Bio-1Kb™ as DNA ladder (New England BioLabs) and ABMM1 phage DNA as positive control. The original gel is presented in Supplementary Fig. S14.

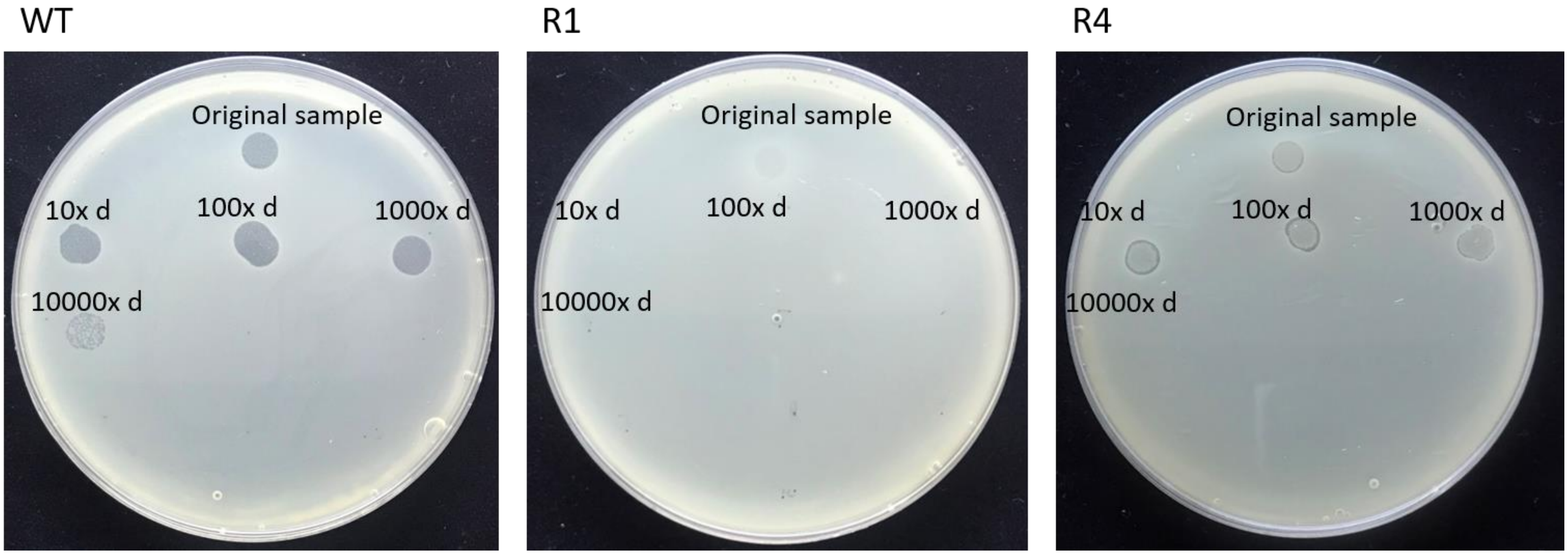

Supplementary Figure S5. R4 lost its resistance to ABMM1 after 5 times of subculture.

Spot test of serial dilution of ABMM1 in WT, R1, and R4 lawn for after 5 times of subculture. WT indicates TV2199.

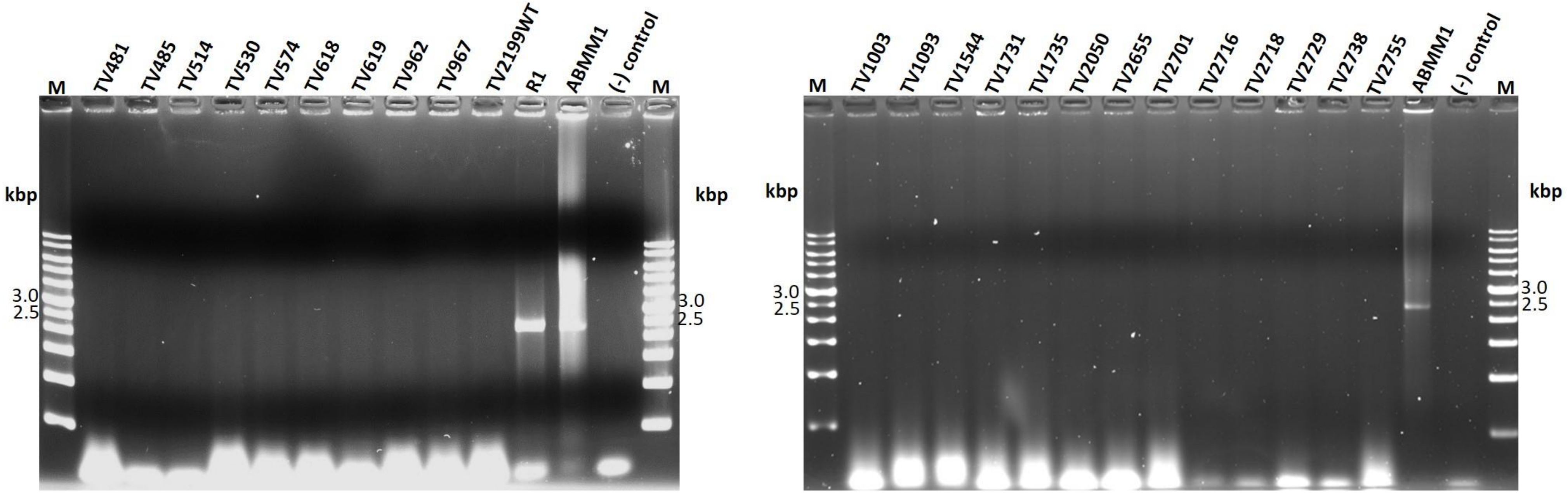

Supplementary Figure S6. Detection of the prophage-like ABMM1 in ABMM1-insensitive *A. baumannii* clinical strains by colony PCR with ABMM1-specific primers (Gp73).

Colony PCR was done using a specific primer for ABMM1 Gp73. The absence band of ~2,3 kbp was shown in 23 strains that do not sensitive to ABMM1 infection (from host range analysis data), while the band appeared in R1 strain, meaning that ABMM1 is integrated into phage-resistant strains (R1) after co-cultivation, while those insensitive clinical strains were not because they carry prophage-like ABMM1. ABMM1 phage DNA was used as positive control, no template added as negative control. Lane M: Bio-1KB^{TM} as DNA ladder. The original gels are presented in Supplementary Fig. S15 and Supplementary Fig. S16 for left and right gel, respectively.

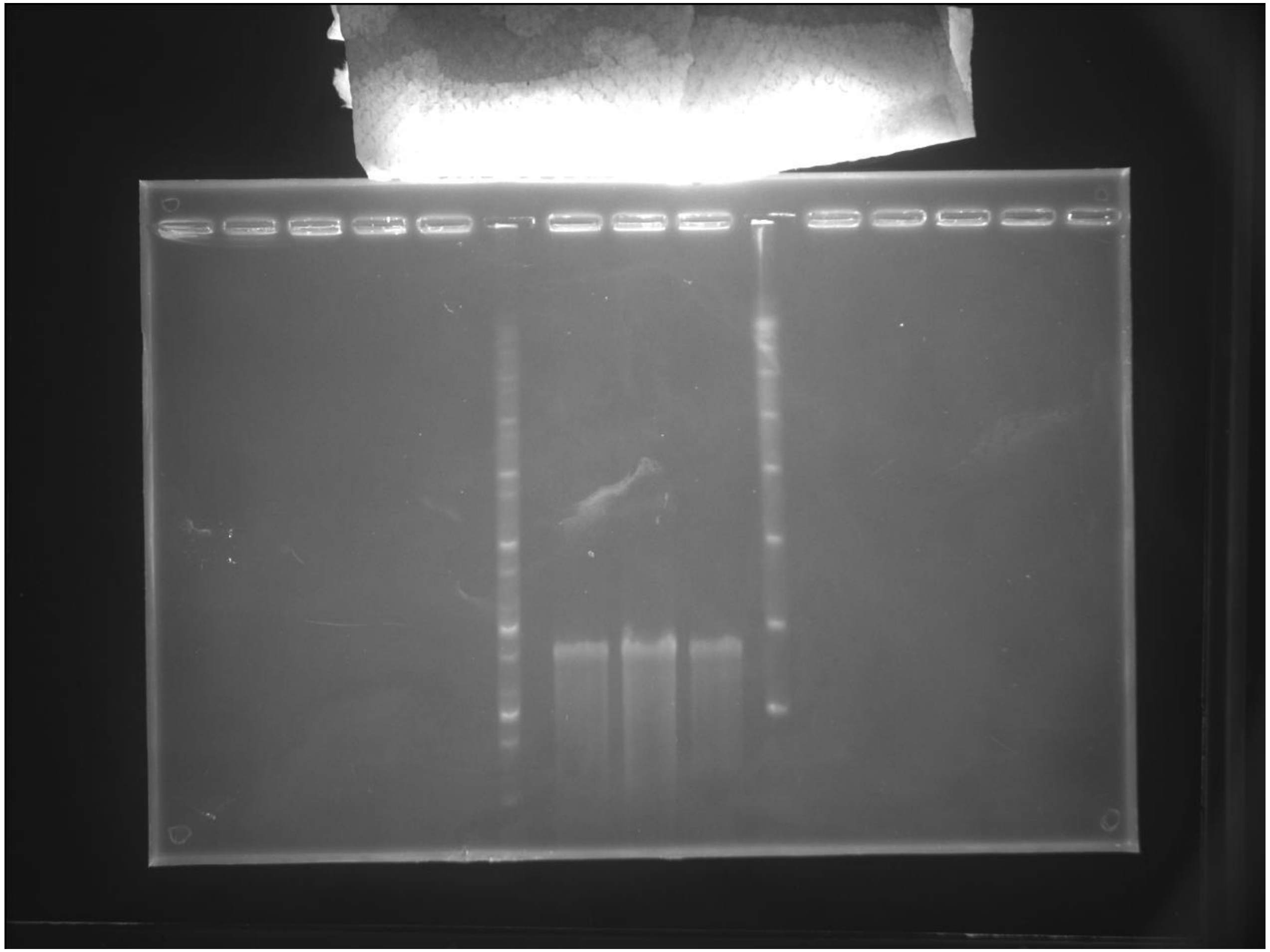

Supplementary Figure S7. The original gel of Fig. 4a.

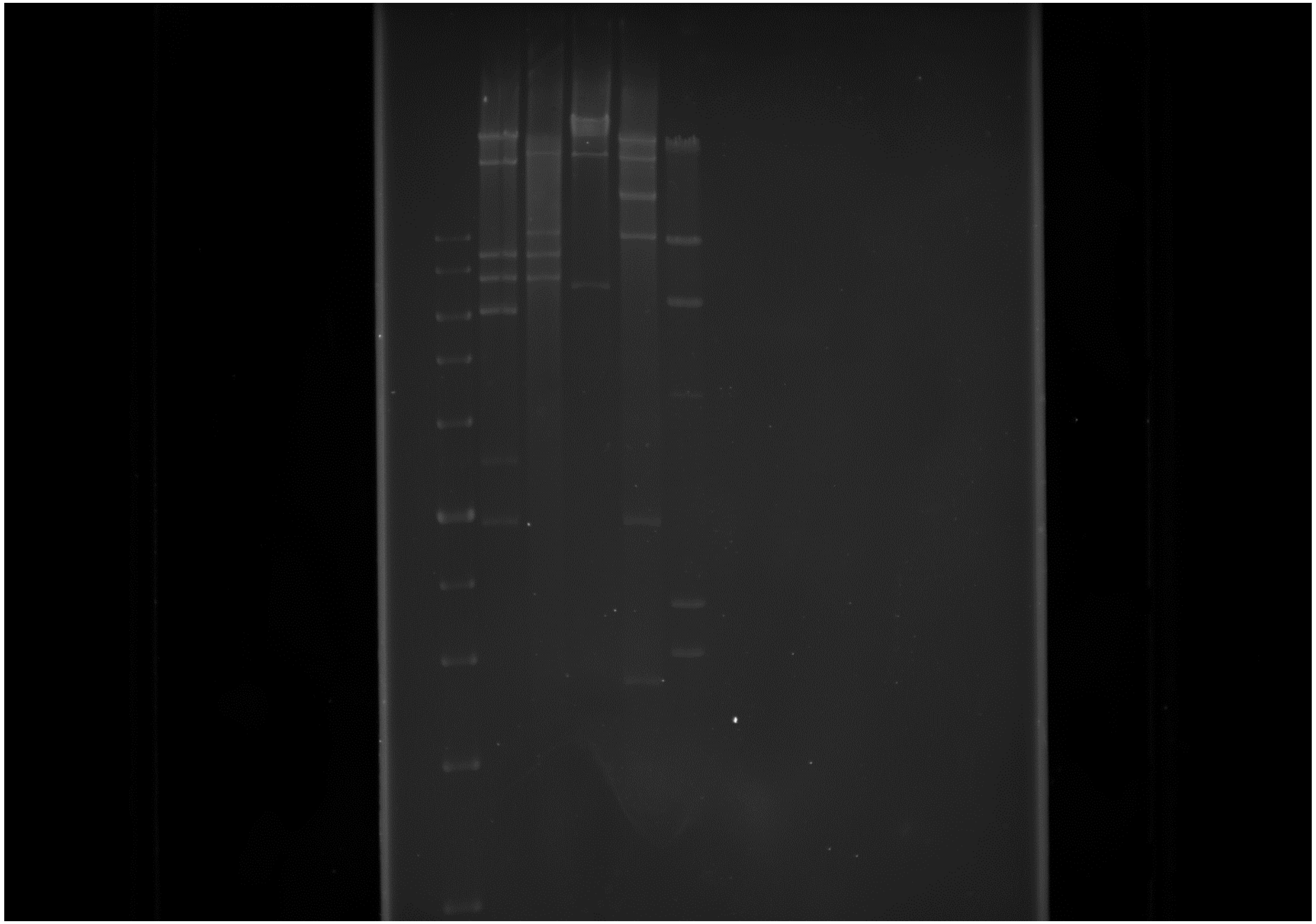

Supplementary Figure S8. The original gel of Fig. 4c.

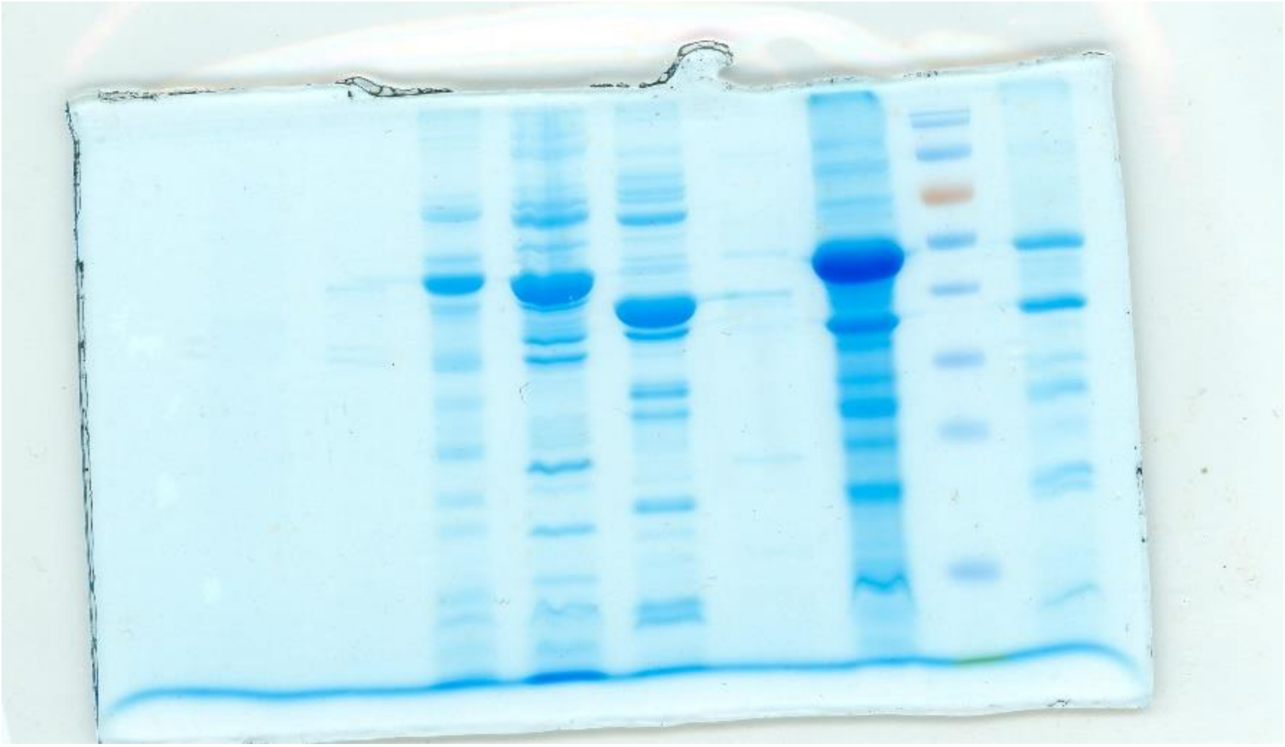

Supplementary Figure S9. The original gel of Fig. 4d.

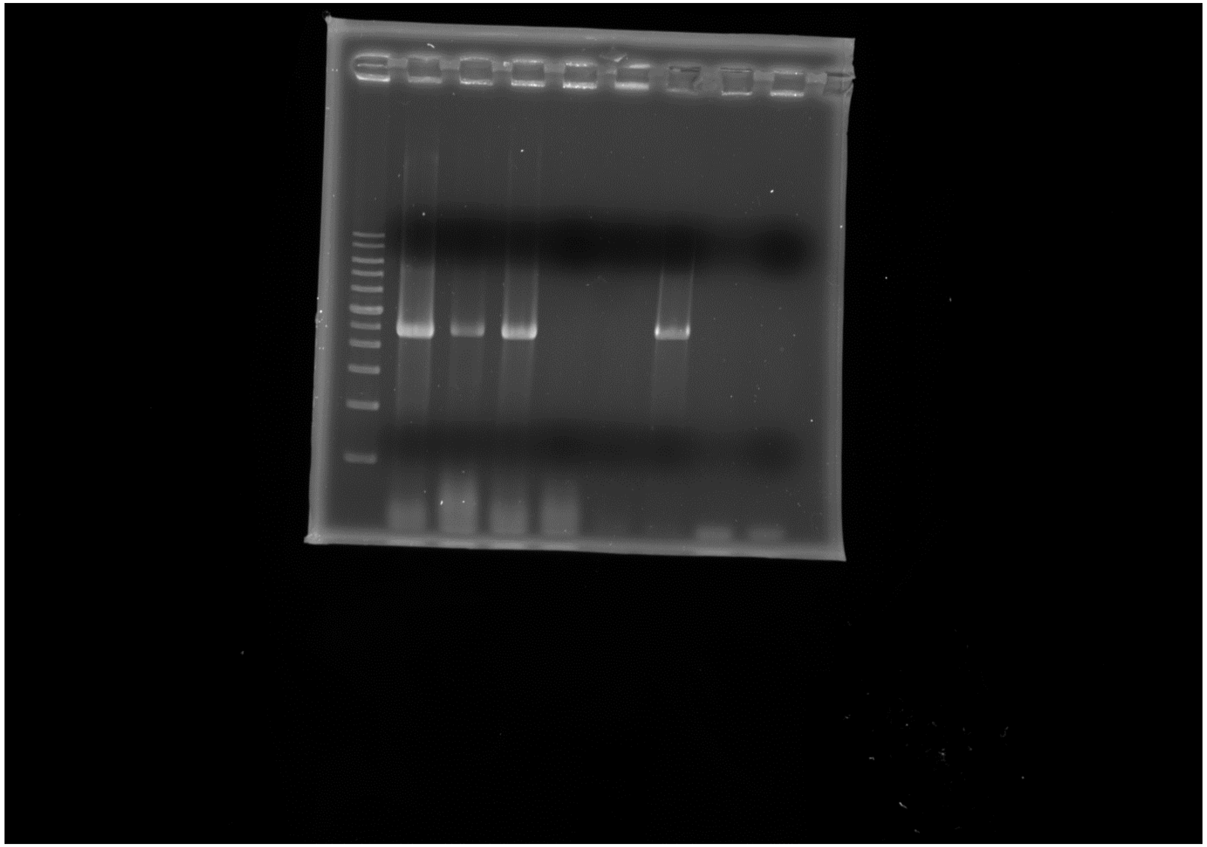

Supplementary Figure S10. The original gel of Fig. 5a.

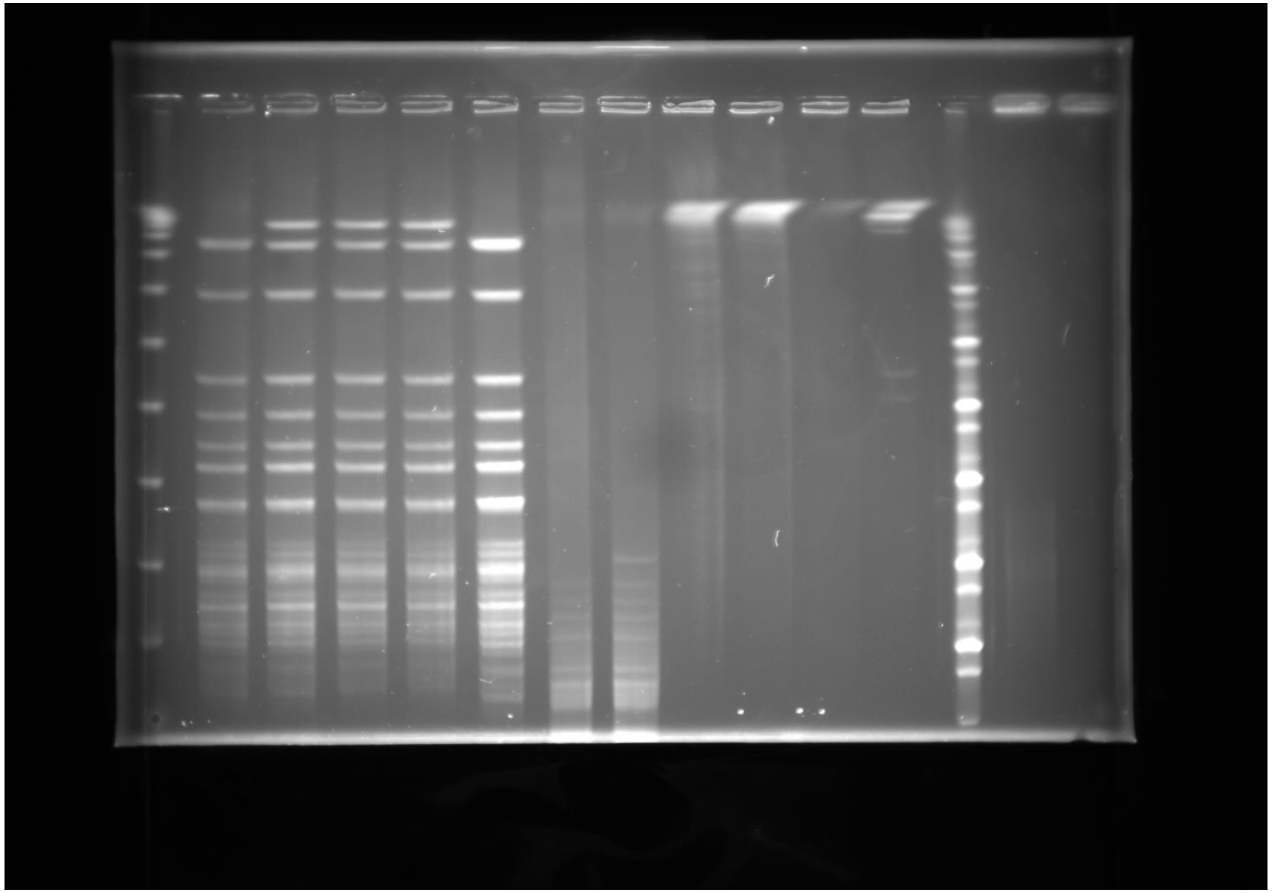

Supplementary Figure S11. The original gel of Fig. 5b.

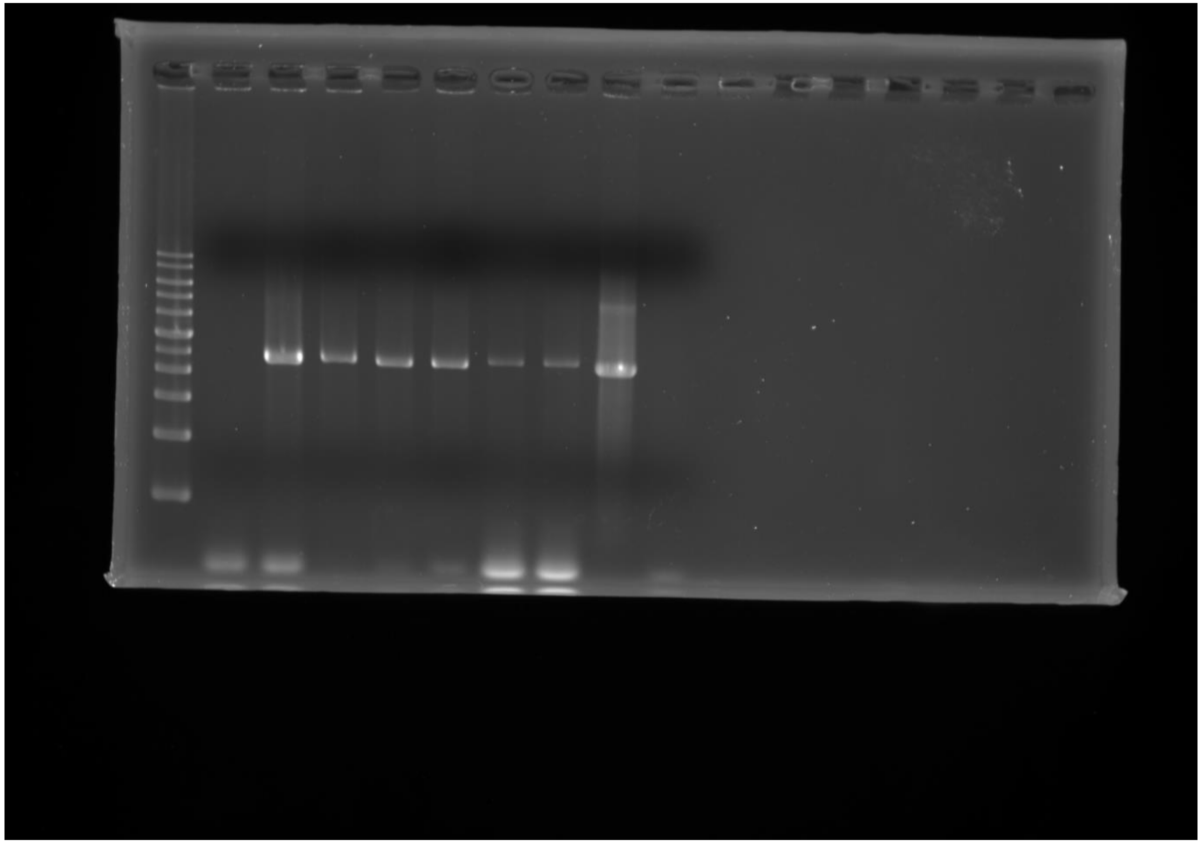

Supplementary Figure S12. The original gel of Fig. 5c.

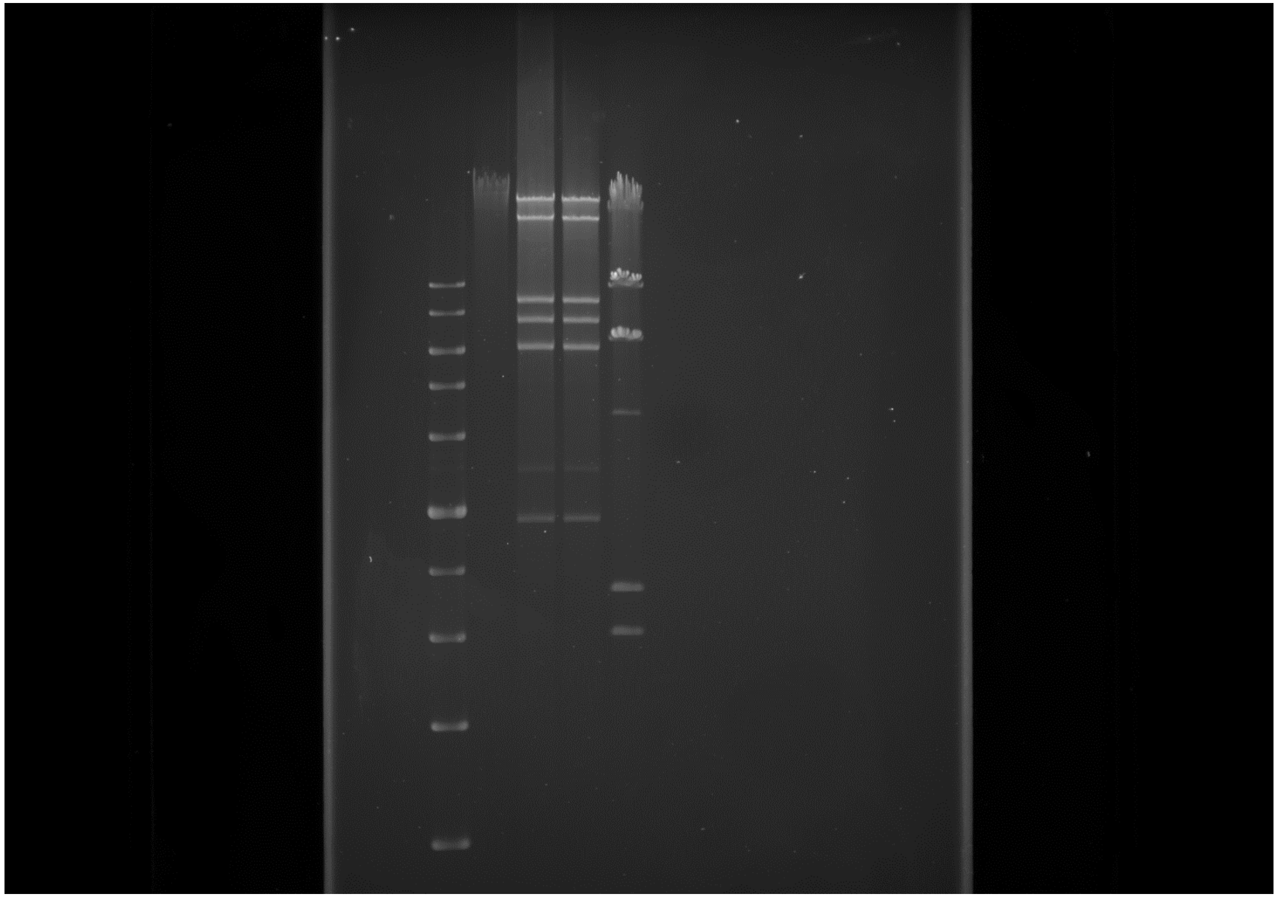

Supplementary Figure S13. The original gel of Fig. S1.

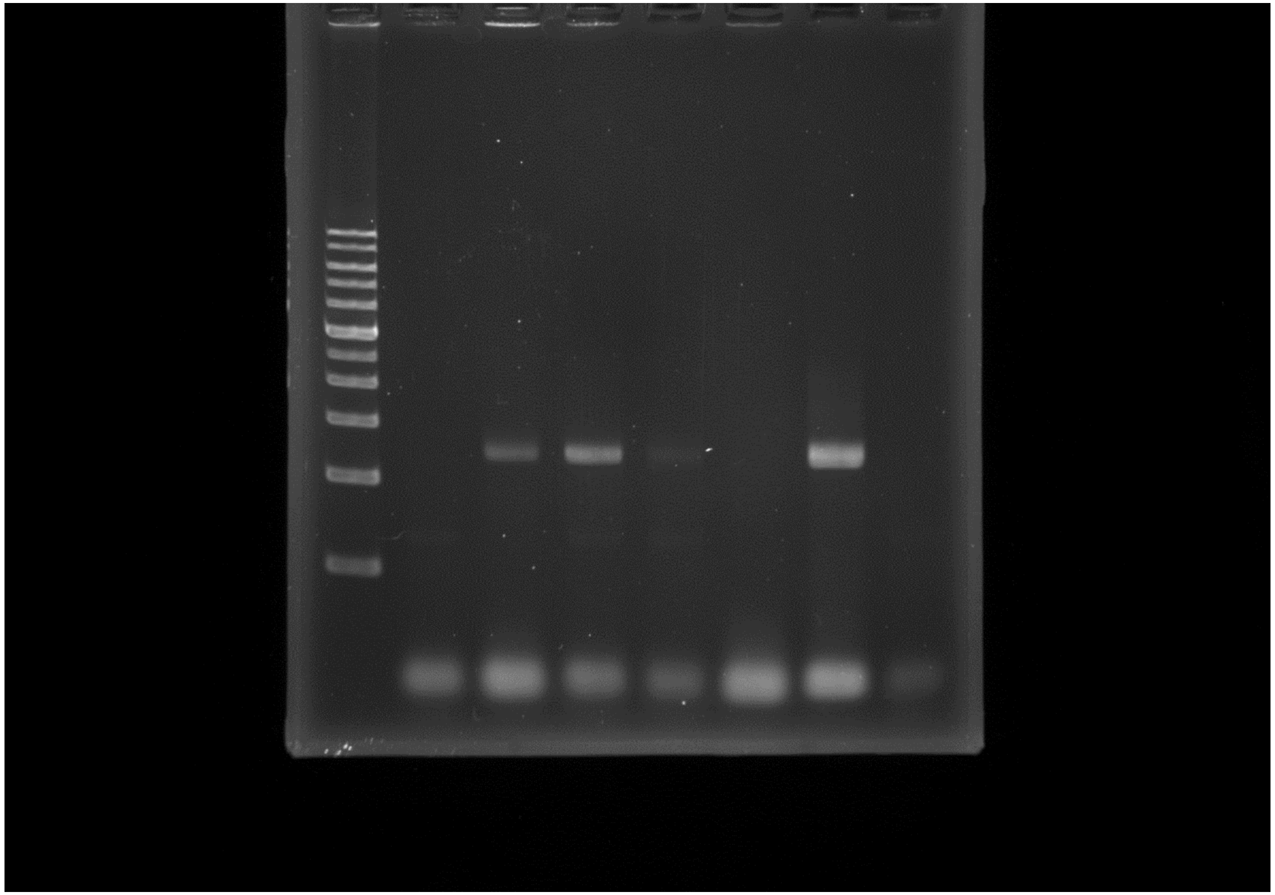

Supplementary Figure S14. The original gel of Fig. S4.

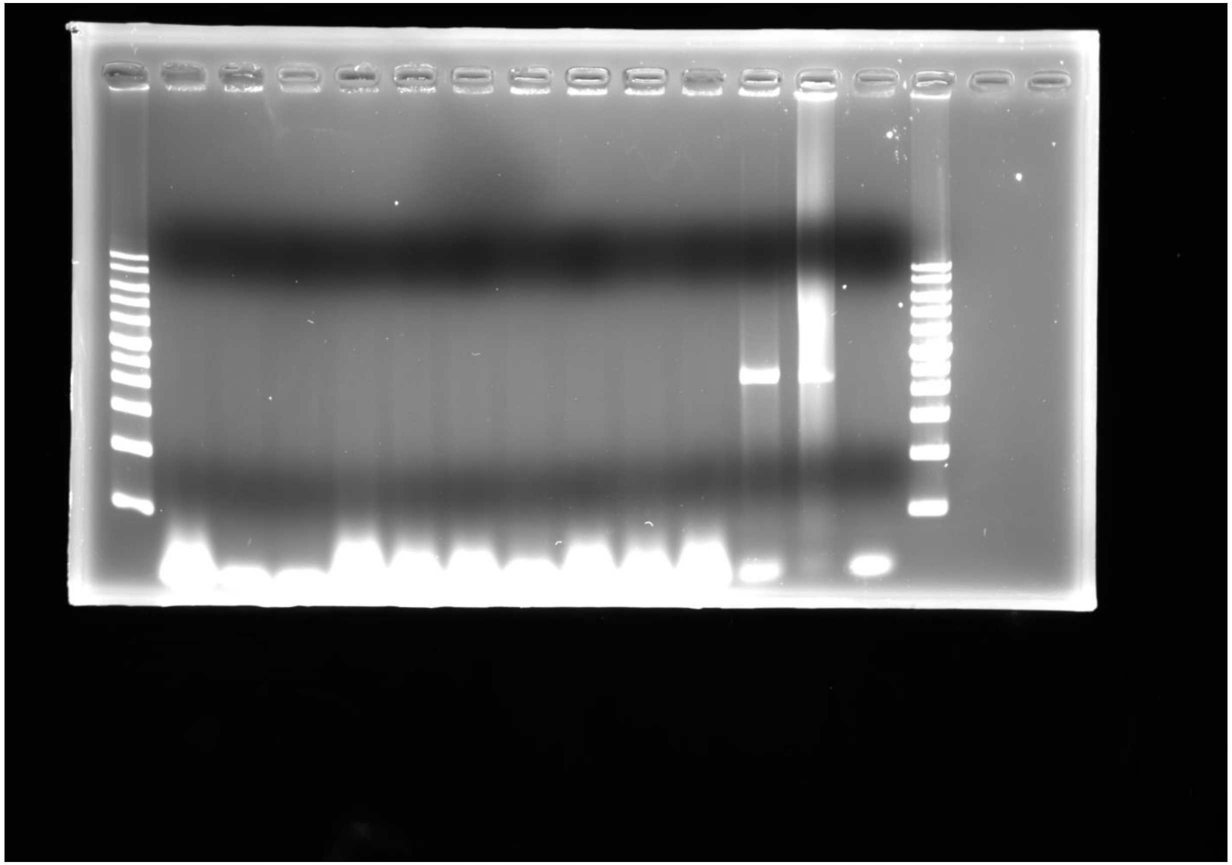

Supplementary Figure S15. The original gel of Supplementary Figure S6 (left gel).

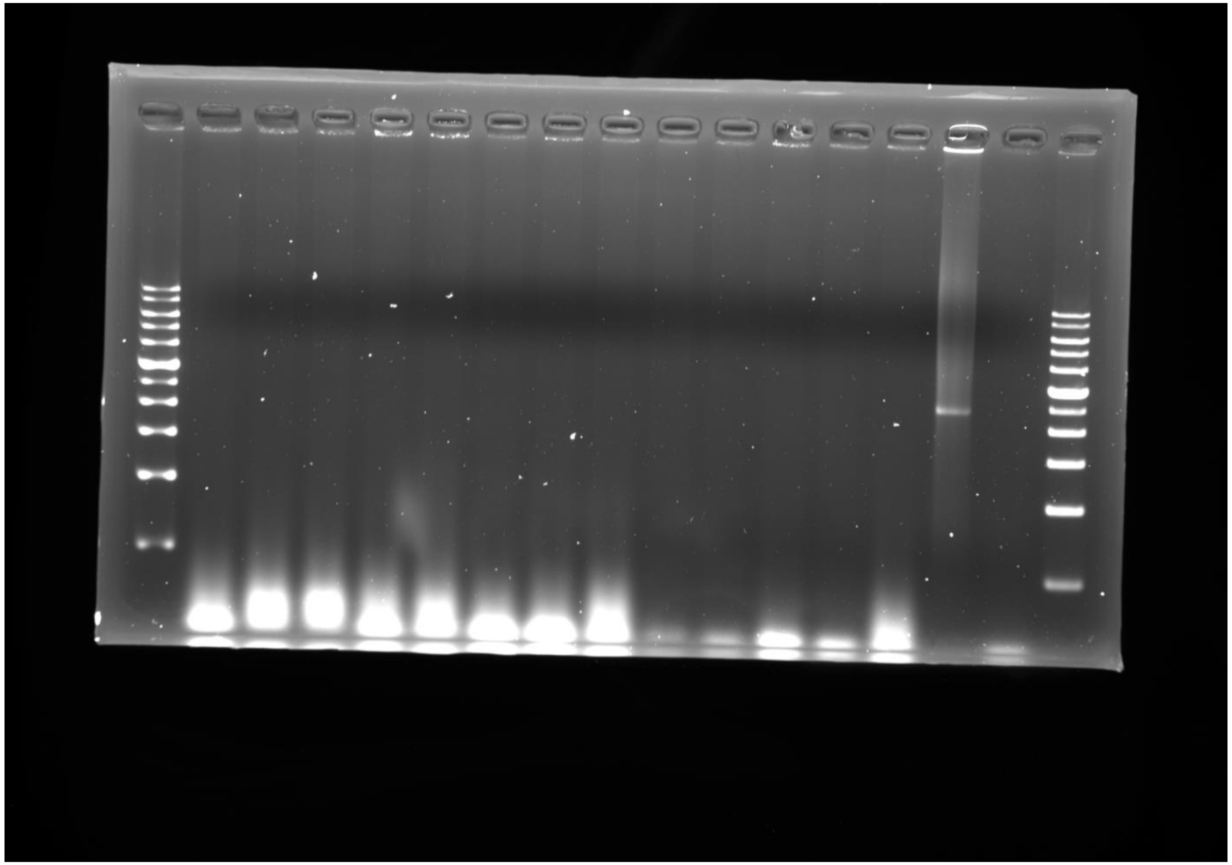

Supplementary Figure S16. The original gel of Supplementary Figure S6 (right gel).
